# Cholesterol and sterols in molluscan endocrinology: past, present, future

**DOI:** 10.3389/fendo.2025.1627166

**Published:** 2025-07-31

**Authors:** István Fodor, Gabriel V. Markov, Luis Alfonso Yañez-Guerra, Károly Elekes, Edit Pollák, László Molnár, Zsolt Pirger

**Affiliations:** ^1^ Ecophysiological and Environmental Toxicological Research Group, HUN-REN Balaton Limnological Research Institute, Tihany, Hungary; ^2^ Sorbonne Université, CNRS, Laboratoire de Biologie Intégrative des Modèles Marins, LBI2M, Roscoff, France; ^3^ Institute for Life Sciences, University of Southampton, Southampton, United Kingdom; ^4^ Department of Neurobiology, Institute of Biology, Faculty of Natural Sciences, University of Pécs, Pécs, Hungary

**Keywords:** mollusks, cholesterol, sterols, synthesis, transport, metabolism, sex steroids

## Abstract

Recently, critical evaluations have challenged the presence of vertebrate-type sex steroid signaling in mollusks, underlying the need of new research lines to shed light on molluscan endocrinology. The investigation of cholesterol metabolism and the potential biological role of sterols in mollusks has emerged as a growing research field in recent years. However, there is no clear consensus on several aspects of this topic and there is a great lack of relevant molecular data. In this perspective paper, we present an overview of our current knowledge on the role of cholesterol and sterols in mollusks and try to outline possible future research directions in molluscan endocrinology. Our study also provides a framework for studying cholesterol synthesis, uptake, transport, and metabolism in mollusks.

## Introduction

Cholesterol is the principal sterol in all animals, playing a key role in various biological processes, including the regulation of membrane fluidity, mediation of intercellular communication, and serving as a precursor for sex steroid synthesis (1). There has been a long-standing interest in the presence and role of cholesterol in mollusks, particularly due to the belief, over many years, that they use the same sex steroids as those of vertebrates to control reproduction. However, this belief has been challenged. There is also no clear consensus on the synthesis, transport, and metabolism of cholesterol, or of the exact biological roles of cholesterol or any other sterols that have been found in mollusks. The process of cholesterol transport mechanisms in mollusks has also not been studied enough at the molecular biological level.

In this perspective paper, we present a concise overview of our current knowledge on the role of cholesterol and sterols in mollusks. Additionally, using the great pond snail (*Lymnaea stagnalis*), a well-established molluscan model in neuroscience and neuroendocrinology [reviewed by (2)], we try to provide a framework for studying cholesterol synthesis, uptake, transport, and metabolism in mollusks. Our aims are 1) to further support the paradigm shift away from the currently prevailing vertebrate-centric view of molluscan reproduction and 2) to outline possible future research directions in molluscan endocrinology.

## Current knowledge on the role of cholesterol and sterols in molluscan endocrinology

### Presence and synthesis

**Figure 1:**
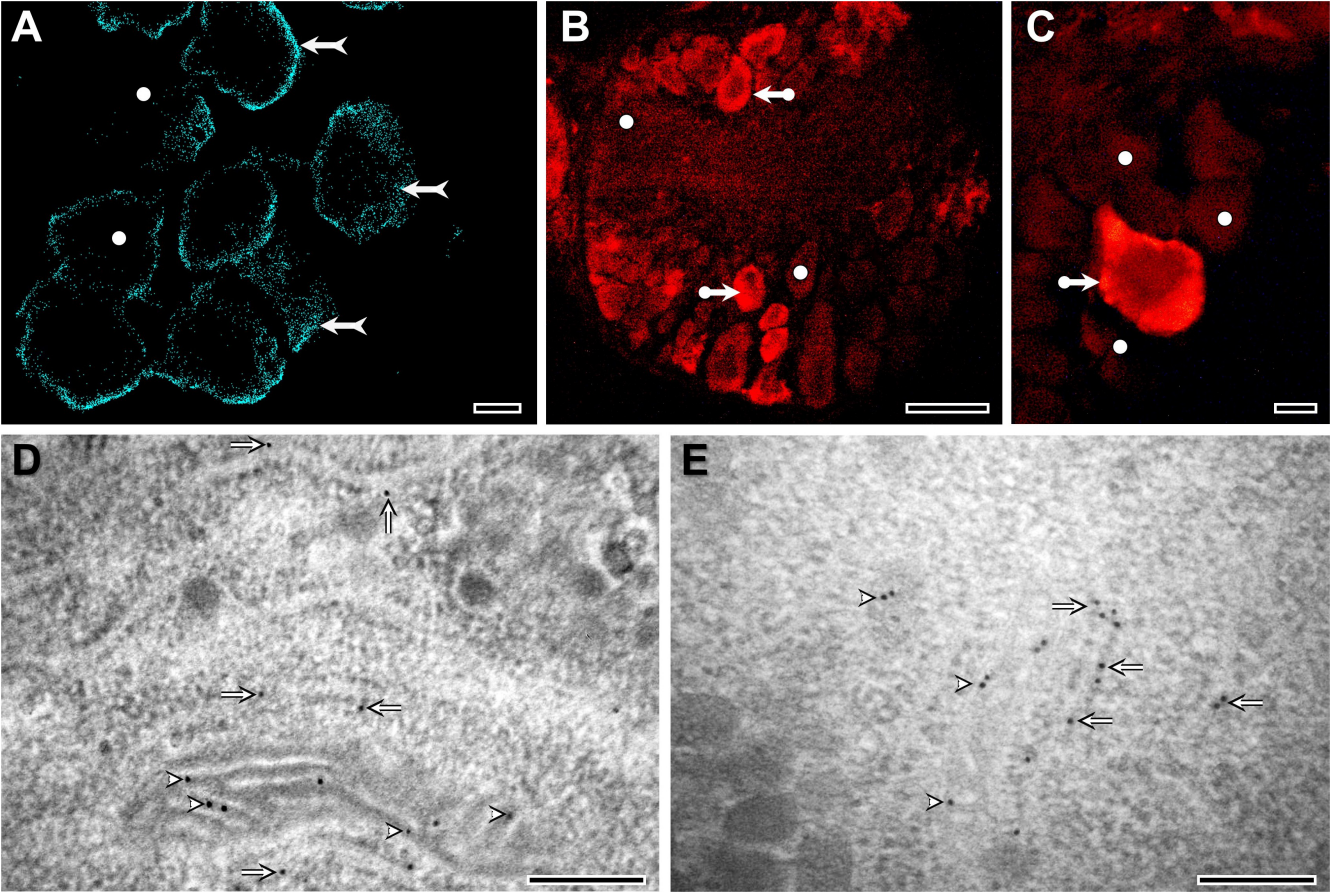
Distribution and localization of cholesterol in *L. stagnalis* neurons, visualized by Filipin III staining **(A)**, immunohistochemistry **(B, C)**, and immunogold labeling **(D, E)**. At the light microscopic level, most of the staining is located close to the plasma membrane (forked arrows) **(A)**. However, the intensity of neuronal staining varies characteristically from cell to cell. Several neurons in the ganglia show moderate or weak labeling (white dots), whereas the majority of neurons exhibit intense immunolabeling (bullet arrows), suggesting differences in cholesterol accumulation/metabolism among neurons **(A-C)**. At the ultrastructural level, the localization of gold particles indicates the presence of cholesterol along the membranes of mitochondria (arrowheads) and rough endoplasmic reticulum (arrows) **(D, E)**. Detailed methodology of Filipin III staining, immunohistochemistry, and immunogold electron microscopy is presented in the **Supplementary Information**. Bars: 10 μm **(A-C)** and 200 nm **(D, E)**.

Cholesterol is present in molluscan tissues in quantities more than sufficient to serve as a precursor for steroid synthesis (3). In *L. stagnalis*, for example, its presence can be demonstrated in all neurons of the central nervous system using histochemical (**Figure 1A**), immunohistochemical (**Figures 1B, C**), or mass spectrometric (4, 5) methods. There is a relatively long-standing debate on whether molluscan species are capable of synthesizing cholesterol *de novo* or whether they rely on obtaining it from their diet. The origin of cholesterol formation, in the endoplasmic reticulum (ER) from acetyl-CoA by a metabolic pathway consisting of more than 20 steps, predates the protostome-deuterostome split and is thought to be broadly conserved across eukaryotes with secondary losses in cnidarians and many protostome taxa (1). Based on the findings of early studies using radiolabeled precursors (reviewed by (6)), the current idea is that the ability to produce cholesterol *de novo* depends on whether the given molluscan species is carnivorous or not. Of course, non-carnivorous mollusks might also rely on obtaining it from their diet: a previous study clearly demonstrated that the northern bay scallop (*Argopecten irradians irradians*) can convert dietary phytosterols into cholesterol through dealkylation (7) – a process well-known in phytophagous insects (8). Interestingly, a recent study showed that, in addition to cholesterol, marine annelids could synthesize sitosterol *de novo* using a non-canonical C-24 sterol methyltransferase (9). Building on this finding, another recent study proposed that although early eumetazoans were capable of synthesizing C28+ sterols, many animal lineages, including mollusks, independently lost their biosynthesis (10). In accordance with this, bioconversion of dietary C28+ sterols to cholesterol has been demonstrated in the larvae of the Pacific oyster (*Crassostrea gigas*), suggesting a contribution of well-conserved and lineage-specific enzymes (11). Importantly, future studies are needed to further characterize the enzymes involved in the bioconversion of dietary sterols and reveal the biological role of such metabolized sterols in mollusks.

### Cellular uptake and transport

Cholesterol is sparingly soluble in water and cannot thus be transported in the bloodstream (or in the case of mollusks, the hemolymph) in the sort of quantities that are needed by the cells of the body. Instead, cholesterol is first of all conjugated to a fatty acid. This is known as esterification and it involves the condensation of the hydroxyl group on the 3^rd^ Carbon atom of cholesterol to the alcohol function of the carboxyl group from a fatty acid with the elimination of a water molecule. The ‘cholesterol ester’ thus formed is then bound up with other fat molecules into spherical particles by lipoproteins. The lipoproteins are amphiphilic and form a shell with their lipophilic ends facing inwards (where the fats are stored) and hydrophilic ends facing outwards. There are two main types of lipoproteins: low-density lipoprotein (LDL) and high-density lipoprotein (HDL).

**Figure 2:**
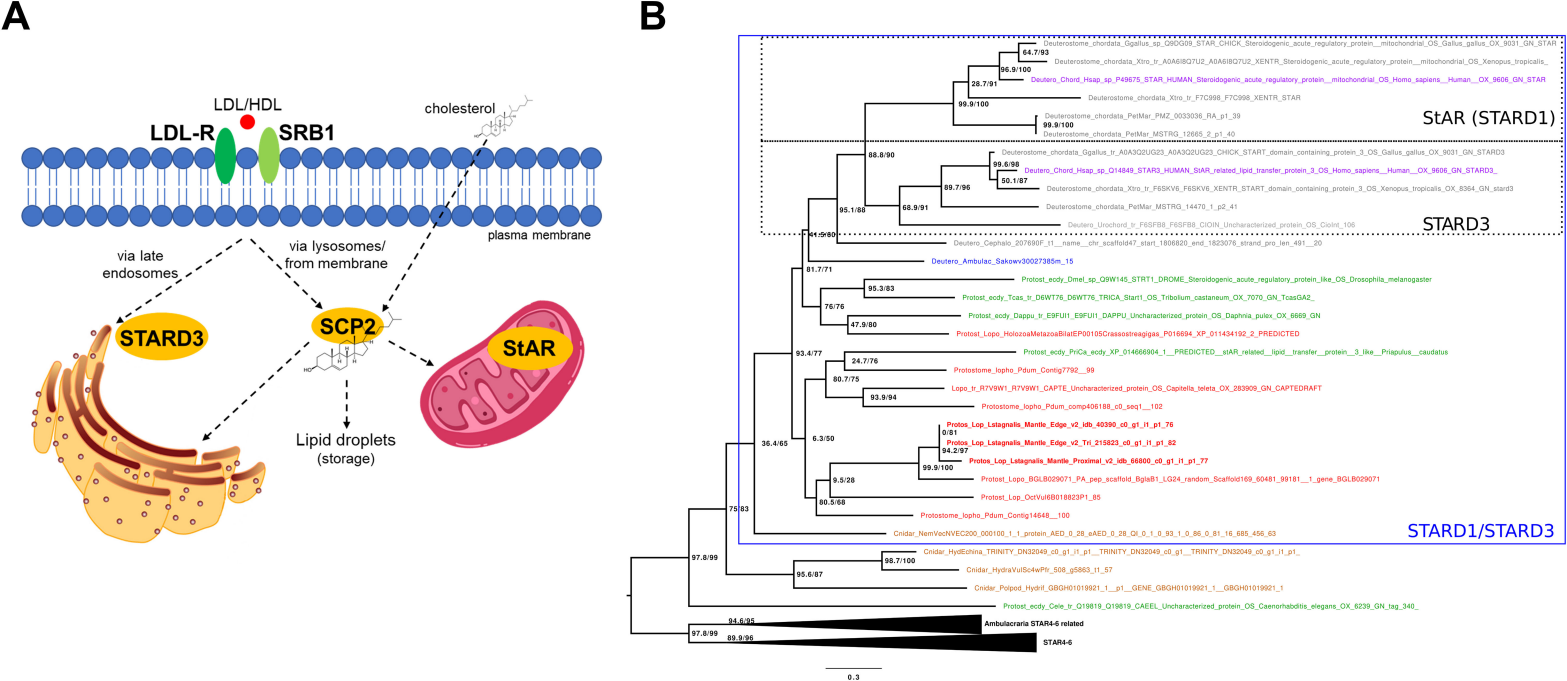
Cellular uptake and transport with the most relevant proteins of cholesterol. **(A)** Schematic overview of cellular uptake and transport of cholesterol in vertebrates. Low density lipoprotein (LDL) binds to the LDL receptor (LDL-R) and is trafficked through the membrane via the classic the endosomal pathway. LDL-containing endosomes fuse with lysosomes, where cholesterol esters are hydrolyzed into free cholesterol which is then distributed by sterol carrier protein 2 (SCP2). Cholesterol esters can also be incorporated directly into the plasma membrane through receptor scavenger receptor class B type 1 (SRB1), a high-density lipoprotein receptor. Once in the membrane, cholesteryl esters are also hydrolyzed and released as free cholesterol. A small percentage of cholesterol can also enter cells via passive diffusion across the plasma membrane. The steroidogenic acute regulatory protein (StAR; STARD1) mediates the transfer of cholesterol between the two membranes of the mitochondria. StAR related lipid transfer domain containing 3 (MLN64; STARD3) mediates the cholesterol transfer at endoplasmic reticulum-endosome contact sites. *L. stagnalis* sequences homologous to these proteins suggest transport mechanisms highly similar to those of vertebrates. **(B)** Phylogenetic tree showing the occurrence and relationships of STARD proteins. The tree shows that StAR and STARD3 represent a chordate-specific expansion. Lophotrochozoans possess many-to-many homologs to chordate StAR and STARD3 proteins. Branch support values are indicated at nodes in the format aLRT/UFBoot (e.g., 100/100). Detailed methodology of the phylogenetic analysis and the raw tree are presented in the **Supplementary Information**.

The uptake of the spherical particles is primarily mediated by LDL membrane receptors (LDL-Rs) on the cell walls (**Figure 2A**) (12), which bind to LDL particles to facilitate their endocytosis into the cell. Once in the cells, they fuse with lysosomes, which contain carboxyl ester lipase 1 (CEL1) enzyme that converts cholesterol ester back into free cholesterol. The free cholesterol is then distributed by sterol carrier protein 2 (SCP2), a non-specific lipid transfer proteins which is also involved in the transport and metabolism of many lipids (13, 14), and utilized in various biological processes. A small percent of cholesterol esters, bound to HDL, can also be incorporated directly into the plasma membrane. This process is mediated by a hydrophobic channel within scavenger receptor class B type 1 (SRB1), a high-density lipoprotein receptor (i.e. without classical endocytosis). Once entering the membrane, cholesteryl esters are also hydrolyzed and then released as free cholesterol. Although SRB1 homologs have been identified in some molluscan species (15, 16), the investigation of cholesterol transporters and carriers in this taxon has somewhat been neglected. Screening both our own and publicly available *L. stagnalis* genome and transcriptome data revealed sequences homologous to vertebrate LDL-R, CEL1, SRB1, and SCP2 proteins (see **Supplementary Information**), suggesting an uptake mechanism highly similar to that of vertebrates.

The intracellular transport of cholesterol within organelles has been of particular interest in molluscan research due to its presumed role as a precursor for vertebrate-type sex steroids (6, 17). In vertebrates, the crucial step in steroid synthesis takes place within the mitochondria, where Cytochrome P450 (CYP) 11A converts cholesterol to pregnenolone (17). Since cholesterol cannot simply diffuse across the aqueous phase between the two membranes, its transfer is mediated by the steroidogenic acute regulatory protein (StAR; also known as STARD1), which is the rate-limiting step in the synthesis of steroid hormones in vertebrates (**Figure 2A**) (13).

Approximately 30% of cholesterol taken up by LDL-Rs is directly transported to the ER (18). In the case of the LDL-containing endosomes targeting the ER, StAR related lipid transfer domain containing 3 protein (STARD3; also known as MLN64) mediates the cholesterol transfer at ER–endosome contact sites (18). Although the presence of StAR has been hypothesized in mollusks in general (1), according to our best knowledge, previous studies have identified only homologs of STARD3 (19, 20). Moreover, there is a single preliminary phylogenetic analysis providing only the information that molluscan STARD3 sequences are separated from vertebrate STARD3s (20). Using Hidden Markov Models, our thorough searches in the available genome and/or transcriptome data of deuterostome, protostome, and non-bilaterian animals revealed candidate genes for both StAR and STARD3. Then, using all-vs-all cluster-based methodologies (**Supplementary Figure S1**) and phylogenetic analysis (**Figure 2B**; **Supplementary Figure S2**), we clearly demonstrated that 1) StAR and STARD3 are a duplication that happened in chordates and that 2) lophotrochozoans have many-to-many homologs to chordate StAR and STARD3 proteins. In *L. stagnalis*, specifically, our analysis revealed the presence of three STARD1/D3 homologs (see **Supplementary Information**). Conserved domain analysis confirmed that these proteins contain the START/SRPBCC domain characteristic of STARD proteins (see **Supplementary Information**). Since our immunogold electron microscopy investigations demonstrated the presence of cholesterol in the inner mitochondrial membrane of *L. stagnalis* neurons (**Figures 1D, E**), we propose that the transport mechanism of cholesterol across the mitochondria in mollusks is similar to that in vertebrates and is mediated by at least one STARD1/D3 protein.

Given that there is no clear evidence for vertebrate-type sex steroid synthesis in mollusks (6, 17, 21, 22), the actual role of cholesterol found within the mitochondria in these species remains unclear. This is also the case for arthropods, as the cholesterol to 7-dehydrocholesterol conversion in the first step of ecdysone synthesis is implemented in the ER (23). Hence, it would also be important to investigate the proteins which mediate the cholesterol transfer at the ER–endosome contact site. In addition to STARD3, proteins such as Niemann-Pick intracellular cholesterol transporter 1 (NPC1), NPC2, Oxysterol-binding protein–related protein 5 (ORP5), ORP1, VAP proteins, Ras-related protein Rab-7a (Rab7), and Rab-interacting lysosomal protein (RILP) are known to play key roles in the process in vertebrates (18). The presence of molluscan homologs to these proteins has been scarcely studied so far (1). Screening the *L. stagnalis* genome and transcriptome data revealed sequences homologous to these vertebrate proteins, except for RILP (see **Supplementary Information**). These homologs, as well as the presence of cholesterol along the membranes of rough ER in *L. stagnalis* neurons (**Figures 1D, E**), suggest a transport mechanism sharing some similarities to that of vertebrates. Future research on other species is required to clarify the general application of cellular processes involved in cholesterol uptake and transport in mollusks.

### Metabolism: a diversity of long-chained steroids as a new direction

A recent study suggested that paraestrol A, an aromatized sterol (i.e. a cholesterol metabolite that has not undergone side-chain cleavage), functions as an activator of the ancestral steroid receptor (22). Given this, investigating sterols as potential hormones in mollusks has been proposed as a new research direction that could shed light on molluscan (21).

Aromatized long-chained steroids (e.g., geodisterol) were first identified unequivocally in sponges and cnidarians (24, 25), and some of them, like the fevicordins, are also found in some land plants [cucurbids and malvales (26)]. This raises the possibility that some aromatized sterols could be present in mollusks as well – with their aromatization being catalyzed by a (still unknown) paralogous CYP enzyme. All those molecules indicate that the chemical space to be explored is larger than the one restricted to vertebrate-type steroids. Moreover, natural product chemists have already identified some specific molluscan sterols. For example, epidioxysterols were identified in *Aplysia* (some of them were isolated from the eggs) (27), referring to the potential role of non-aromatized steroids in mollusks, either as defense compounds or as hormones. We propose that molluscan 5α-reductases are involved in the synthesis of such sterols. 5α-reductase is an ancient enzyme present in both plants and animals and is known to use sterols (as well as testosterone and androstenedione in vertebrates) as substrates (28, 29). In plants, campesterol is a substrate for 5α-reductase (30), so it is reasonable to suppose that molluscan 5α-reductases could catalyze a similar reaction on cholesterol. This would make perfect sense when looking at the structures of the 5α,8α epidioxysterols reported in *Aplysia*: those molecules derive probably from cholesterol, and they must lose the delta 5–6 double bond by delta-5-reduction at some point. The potential role of sterols in molluscan endocrine processes is also supported by a recent finding that inhibition of 5α-reductase caused marked shell malformations during the embryonic development of freshwater gastropods (30). Additional to epidioxysterols, other secosteroids, which may derive from further oxidative degradation of the sterol backbone, were also identified (27), indicating that the chemical space to be explored is large, and enabling a variety of functions for all those molecules. An additional important question is in which tissues these steroids are synthesized in mollusks. Previous preliminary analyses conducted in the Aplysia genus have shown that these metabolites are not accumulated in a specific organ but distributed in the whole organism (31). However, their presence in a given tissue does not necessarily imply that synthesis occurs there. Tissue-specific gene expression studies (e.g., analysis of the tissue distribution of the 5α-reductase enzyme) are required to clearly determine the production sites. In summary, future studies, on the one hand, are needed to further characterize the diversity of molluscan sterols, and on the other hand, to identify the molluscan enzymes and tissues involved in sterol synthesis.

It is worth noting, however, that the concept of sterols as potential hormones warrants careful consideration in light of their chemical nature. Sterol-based compounds, like paraestrols, share structural similarities with cholesterol, which is characterized by its low water solubility. Due to this property, sterols are generally not freely soluble in aqueous environments such as the hemolymph and are therefore unlikely to be efficiently transported as classical circulating hormones. Instead, their bioactivity is likely confined to the cells or tissues where they are synthesized, suggesting a role more consistent with autocrine or paracrine signaling rather than endocrine function. This physicochemical constraint highlights the need for caution when applying the term “hormone” to sterol-like molecules, as their mode of action may not involve systemic transport but rather localized, cell-autonomous effects.

Finally, it is possible that some molluscan long-chain steroids bind to nuclear receptors belonging to the NR1H or NR1I/J/K families, which include oxysterol and bile acid receptors in vertebrates, the ecdysone receptor in arthropods, and the dafachronic acid receptor in nematodes. In our opinion, this is the direction where it would be reasonable to search for nuclear receptor-based steroid signaling in lophotrochozoans (see also (32)). Speculatively, long-chained steroids might also exert their effects via non-genomic signaling, through either membrane receptors or other lipid-binding domains which are not directly transcription factors. Indeed, long chained vertebrate bile acids were found to be activators of sodium channels in an acorn worm and a brachiopod, suggesting that unknown bile acid-like molecules may be the endogenous ligands for those receptors that are widespread across bilaterians (33).

## Summary

Since the 1950s, a vertebrate-centered perspective has characterized molluscan endocrinology research. Starting from 2011, critical evaluations have challenged the concept of sex steroid signaling in mollusks, highlighting the absence of endogenous synthesis and nuclear receptors for vertebrate-type sex steroids (6, 17, 21, 34–37). In light of this, clearly, new research directions are needed to shed light on actual questions in molluscan endocrinology. We believe that investigation of sterols can significantly contribute to the understanding of molluscan endocrine systems, such as nuclear receptor-based steroid signaling, although not necessarily only in relation to reproduction. Future studies should aim 1) to investigate cholesterol metabolism and sterol synthesis in details, 2) to identify sterols and their receptors in different molluscan species, and 3) to investigate the role of sterols in molluscan physiology.

## Data Availability

The datasets presented in this study can be found in online repositories. The names of the repository/repositories and accession number(s) can be found in the article/**Supplementary Material**.
