# Supplementary material for: Cholesterol and sterols in molluscan endocrinology: past, present, future
Source: Front Endocrinol (Lausanne). 2025 Jul 31;16:1627166. doi: 10.3389/fendo.2025.1627166 (PMC12350143; doi:10.3389/fendo.2025.1627166)
Supplement: Supplementary file 1 [file DataSheet1.doc]

**Supplementary information**

**Cholesterol and Sterols in Molluscan Endocrinology: Past, Present, Future**

^1^István Fodor*, ^2^Gabriel V. Markov, ^3^Luis Alfonso Yañez-Guerra, ^1^Károly Elekes, ^4^Edit Pollák, ^1^László Molnár, ^1^Zsolt Pirger

^1^Ecophysiological and Environmental Toxicological Research Group, HUN-REN Balaton Limnological Research Institute, Tihany, Hungary

^2^Sorbonne Université, CNRS, Laboratoire de Biologie Intégrative des Modèles Marins, LBI2M, F-29680 Roscoff, France

^3^Institute for Life Sciences, University of Southampton, Southampton, United Kingdom

^4^Department of Neurobiology, Institute of Biology, Faculty of Natural Sciences, University of Pécs, 7624 Pécs, Hungary

*Corresponding author; E-mail: [fodor.istvan@blki.hu](mailto:fodor.istvan@blki.hu)

**Experimental animals**

5-month-old adult, mature specimens of *Lymnaea* were obtained from our laboratory-bred stocks (HUN-REN BLRI). Snails were maintained in large plastic tanks containing 10 L oxygenated artificial snail water (composition in mM: 0.1309 NaHCO_3_, 0.0378 K_2_SO_4_, 0.4013 CaCl_2_^.^2H_2_O, 0.0390 Mg(NO_3_)_2_^.^6H_2_O; pH=7.6) at 20 °C (±1 °C) on a 12:12 h light:dark regime with natural wavelength light. Specimens were fed on lettuce *ad libitum* two times a week.

**Ethics statement**

All procedures, methods, and experiments on the specimens were carried out in accordance with the relevant guidelines and regulations approved by the Scientific Committee of Animal Experimentation of the Balaton Limnological Research Institute (VE-I-001/01890-10/2013).

**Sample preparation for light and electron microscopy**

The whole CNS (n = 15) were dissected from the animals and immediately immersed in freshly prepared ice-cold fixatives according to the goal of the investigations. For Filipin III staining, the applied fixative was 4 % paraformaldehyde in 0.1 M phosphate buffer (PB, pH = 7.6). For immunofluorescence and immunogold labelling, a mixture of 4% paraformaldehyde, 0.25 % glutaraldehyde, and 0.2 % magnesium chloride in 0.1 M PB (pH = 7.4) was used. Following overnight fixation, samples were thoroughly washed (6 changes) with phosphate-buffered saline (PBS) and used for either light or electron microscopy.

For light microscopy, the samples (n = 10) were immersed in 15% sucrose solution (in PBS) for an hour, then with 30% sucrose solution overnight at room temperature, and embedded into cryomatrix (#6769006, Thermo Scientific). A series of Serial sections with 15 µm cryostat sections were cut and thaw-mounted onto Superfrost Ultra Plus slides (Thermo Scientific). After removing the cryomatrix with washing of freshly prepared 0.1 M PB containing 0.15 % glycine (three changes for 15 mins), the sections were stained for cholesterol labelling.

For electron microscopy, the samples (n = 5) were fixed in ice-cold 1 % osmium tetroxide in PBS for 2 hours, washed, dehydrated with an ascending series of ethanol, and immersed in propylene oxide and embedded into Durcupan ACM resin (Merck, Hungary). Ultrathin sections were cut with a Reichert ultramicrotome equipped with a diamond knife and were collected onto nickel grids.

**Filipin III staining**

The sections (n = 5) were incubated with freshly prepared Filipin III (#F4767, Merck) solution (0.05 mg/mL in PB containing 10% fetal bovine serum) for 2 hours at room temperature. Preparations were thoroughly washed with 0.1 M PBS, then coverslipped and observed with a TCS SP8 DMI laser confocal scanning microscope (Leica Microsystems, Germany) with excitation at 405 nm and emission at 385-470 nm. Control sections were not incubated with Filipin III solution and they did not contain labelled structures.

**Immunofluorescence staining**

After a mild permeabilization with 0.25 % Triton X-100 in PBS, the cryostat sections (n = 5) were covered with 300 mL of a rabbit polyclonal anti-cholesterol primary antibody (#CAU25467, Biomatik, USA) diluted in 1:100 in 0.25% BSA in PBS and incubated overnight at room temperature in humidity chamber. Then sections were thoroughly washed with PBS (six changes for 10 mins) and incubated with an anti-rabbit IgG secondary antibody conjugated with TRITC (#R0270, Dako, Denmark) diluted in 0.25% BSA in PBS at room temperature for 2 hours. After thorough washing with PBS (six changes for 10 minutes), the sections were coverslipped with glycerol-jelly and investigated with a Zeiss fluorescence microscope using TRITC filter cube set. Controls were not incubated with the primary antibody, thus they did not have fluorescent emissions.

**Immunogold labeling**

The resin was etched with 1% periodic acid from the ultrathin sections (n = 5), then they were deosmicated in 1% sodium metaperiodate and washed thoroughly in distilled water (three changes for 5 minutes) and Tris-buffered saline (TBS; 0.1 M, pH = 7.4). After washing, the grids were transferred to 5% normal goat serum for preincubation (30 minutes), followed by incubation with a rabbit polyclonal anti-cholesterol antibody (#CAU25467) diluted 1:50 in TBS overnight in a humidity chamber. After washing with TBS and 1% normal goat serum, the grids were put on a drop of an anti-rabbit IgG secondary antibody (1:25 in TBS) conjugated with 12 nm colloidal gold (Jackson Immunoresearch). Finally, the samples were washed in distilled water and counterstained with uranyl acetate and lead citrate. Controls were performed by omitting the primary antibody resulted in no immunostaining.

**Methodology to identify STARD proteins in Lymnaea** **stagnalis and other species**

STARD protein sequences were identified using Hidden Markov Models (HMMs). Reviewed STARD sequences were retrieved from the InterProScan entry IPR000799, which corresponds to proteins containing Steroidogenic Acute Regulatory (StAR)-like motifs. These sequences were aligned using MUSCLE (automated), and a model was built using hmmbuild with HMMER3. This ad hoc model was then used to search for STARD proteins in the translated transcriptomes of Homo sapiens, Gallus gallus, Xenopus tropicalis, and Petromyzon marinus (Vertebrata); Acanthaster planci and Saccoglossus kowalevskii (Ambulacraria); Branchiostoma belcheri (Cephalochordata); Ciona intestinalis (Urochordata); Daphnia pulex, Drosophila melanogaster, Priapulus caudatus, and Tribolium castaneum (Ecdysozoa); Lymnaea stagnalis, Biomphalaria glabrata, Capitella teleta, Octopus vulgaris, Crassostrea gigas, and Platynereis dumerilii (Lophotrochozoa); Caenorhabditis elegans (Nematoda); and Hydractinia echinata, Hydra vulgaris, Nematostella vectensis, and Polypodium hydriforme (Cnidaria), using an E-value threshold of 1e-5.

The identified sequences were concatenated and analyzed using CLANS (**Supplementary Figure 1**), with BLAST high-scoring pairs (HSPs) extracted at an E-value threshold of 1e-4. A cluster-based approach was used to assess relationships among the proteins with an e-value for connections of 1e-25 using CLANS, and the cluster containing STARD proteins was selected for phylogenetic analysis.


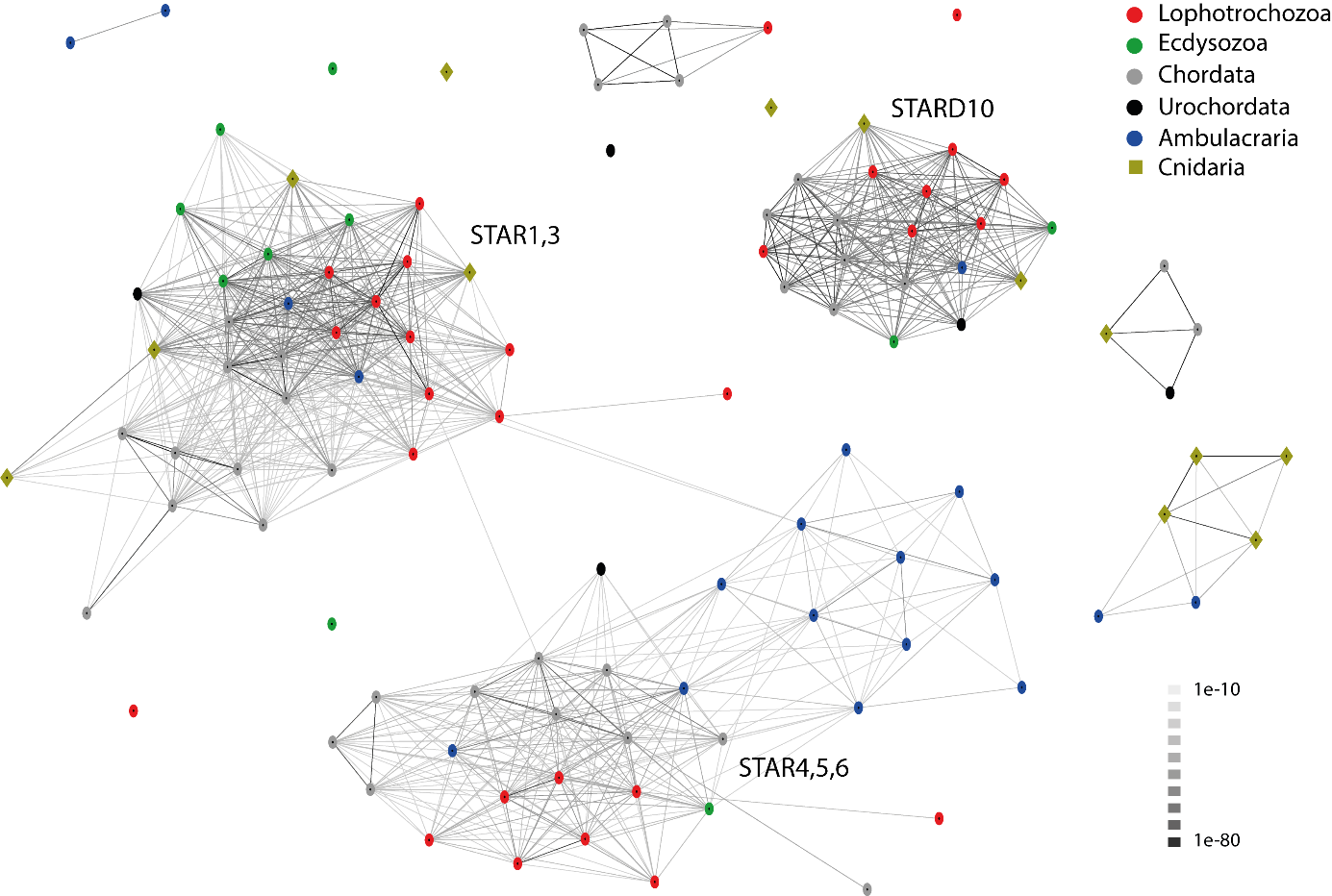


**Supplementary Figure 1**. Cluster map of non-bilaterian, protostome, and deuterostome STARD proteins showing the closely related STARD1,3, STARD4,5,6, and STARD10 proteins.

For the phylogenetic reconstruction (**Supplementary Figure 2**), sequences were aligned using MAFFT version 7 with the iterative refinement method E-INS-I (1). Alignments were trimmed using TrimAl in gappy-out mode (2). Maximum-likelihood trees were inferred using IQ-TREE2 with the LG+G4 model. Branch support was assessed using 1,000 replicates of both the SH-like approximate likelihood ratio test (aLRT) and the ultrafast bootstrap (UFBoot) method (3). Support values are shown on the tree in the format aLRT/UFBoot (e.g., 100/100). The final phylogenetic tree was visualized and annotated using FigTree.





**Supplementary Figure 2**. Raw phylogenetic tree showing the occurrence and relationships of STARD proteins.

**Newly identified *Lymnaea* sequences (mRNA data, protein data, and conserved domains:** <https://www.ncbi.nlm.nih.gov/Structure/cdd/wrpsb.cgi>**)**

1) LDL-receptor 1_NCBI number: PV615401

>mRNA

ATGTCGGATCGAAAAATGCTGTTTAGACAAACAACTATGTTTGTTAAACTTTTTTTGGTAATCGTATTATATGTGGATTCTTCATTTGGGAATAACTCCTCAGATATCACAGATTTAGATTGTGGACCTTCTGAGTTCGCCTGTATTAGTAGTAATAAATGTGTAACTTTGTCTTGGAAATGTGATGGAGACCCTGATTGTCCTGATGGTGAAGACGAGAAAAACTGTTCAGCAACAACATGCACCCCTAATTTTTTCCGGTGCAATAATGGTCTTTGTATATCTGAACGATGGGTGTGTGATGGGCAGGATGACTGCAAGGATAACTCTGATGAAGCAACTGAAGTTTGTGCTGAACGTAATACCTGTGTGAATAAGCACAAATGCTCAGGCACAAATATGTGCATTGACCCTTCATTGGTTTGTGATGGTCAGCCTGACTGCCCAGAAAAAGATGATGAAAAAGATTGCTCAAATCTTGGTTGTGATAAAGAAACTGAATTTAAATGTCATGATAACAATGGCCCTTGCATTTCTTCACGTTGGCATTGTGACGGCTCAGATGACTGCGAGGATGGGTCAGATGAAAAGAATTGCACATCCCAATGTGAGAAAGGTTTTAAGACTTGTGCTGATGGTGAATGCATAAACGATCTCTGGTGGTGCGATGGTGACGCTGATTGTGCCGATAAATCGGATGAAGCAAAGTGTGCTGAATTGGCAGTGAATCAAAATTGCTCAGAAACTGAATTCCAGTGTCAGGGAATGTCTATGGTTGTGCAATGTGTTCATATGAACTGGGTGTGTGACGGCGATTCCGACTGTCTTGATGAATCGGATGAAAAAGATTGTAAACCACATACATGTAACCCGAATGAGATTATTTGTGACAAAAATTTCTGCATCAGTGAACTTTATGTATGCGATGGTGATAAAGATTGTGAGGACGGAACTGATGAGTTTAATTGTACAGTTTCCTGTGATGTGGACAGTTTTGATTGTGGTAATCAGACATGCATCCCCAAAACCCAACTTTGTGATGGTTTTGATCACTGCATTGATGGTGCAGATGAATCCCCATTGCATTGTTCTTCTCAAGTTTCTTCTCAAACTTCATGTGAACACAATAATGGTGGCTGTATGCATTTGTGCAAACCAAATATTAATAGCACAGGCAGGACATGTGAGTGCCGTCCAGGGTATCAGTTAGCCAACCATTCTAATACAGATTGCCAGGATATAAACGAATGTGAGACACCTGGCACATGCTCACAGAAATGTGTCAACACTAAAGGATCTTACAAATGTGACTGTGAACCAGGTTACACATTAATTGTACCTCGTTACTGCAAAGCTATTGAAGGAAAACATGCTGTACTTATCTTGTCCGATAGTCATGAACTTCGTCGTTACAGTTTGGATGAGTTCCGTTATTCCAAGCTTCTTGAAAATCCTGTGCAAAGGGCTGTTGCAATGGATTTTAATATCAGGGAACATAAAGTTTATTGGACAGATCTCCAGCATAAACAAGTTTCTGTGGCTGGTGTAAATGATGTCAATCATATGAAAGTGATAGTTAATGAAAGCATTGTCAAGCATCCAATGAAAGTGATAGTTAATGAAAGCATTGATATGCCGGATGGCCTTGCTGTTGATTGGGTGCATGGTAATTTGTACCTTTCCGACACTGGACTGGACAAAATAGAAGTTTTACGTTTGAATGGCAGTCATCGGAAAATTTTGGTTAATACAGATCTTGATGAACCCAGGGCATTAGTTTTGGATCCACAATACGGATGGATTTACTGGTCAGATTGGGGTGTCCAACCAAAGATTGAGAAGTGCGGGATGAATGGCCAAAATAGGAAATCCATTATCACCAAACATATCGCATGGCCTAACGGTTTGACAATCGATTACATTCAGAGACGCCTTTATTGGGTTGATGCAAAGCTGCACACAATTGGAAGCAGTGACCTTGATGGCAATAACTATAAGTTGATACTGAAGAATCATGGCTACCTTGGCCATCCATTTGGCATAACTGTCTTTGAAGACTACCTCTACTGGACTGACTGGATGACCAATGGTGTTTTCAAATACAGTAAATTTAGTCAGGGCAATGTGACCCACGTAGTCATGAAGCTGAAGACTCCAATGTGTATTCATGCTTATCACGTCGTAAGACAGCCCTCTCCTATGAATCACTGTGGTGACAATAATGGTGGCTGTAGCCACCTATGTCTCCCCAAGCCCCATGAAGGAGTTGTAGCACCCACTACTCCAAACTATGAATGTGCTTGTCCTGATGGAATGAAATTCGGAGAAGGGGAGGAAAAGCACCACTGTGTTCCATCACATGAGGTGGTAACAAATAAAATCCCAACAGATCAAGGGAAAGACATGTCCAATCCAAACCGTTTGCCATACACAATACAGACTCCACCAGAAACACAAACAACAACAAAGTCCTCTTCAAGTCAAGAGACTCAAACAAATTTCAACCAGGGTGAAACAACAGATGGGGTGATCCCTGCGTTAAATGGCACAAATAGTCAGTTGGCACACAAAGAATCTGAAGGAAATGGCAGTGTTGCAATAATTGTCATCGTTATCGTGTTTGTGCTAGGTGTTACTGTCGCTATTATTGTTGCATTGCTGATAAGAAGACATAAGAGGAAAAATGTCAAATCCATGAATTTTGATAATCCAGTATATCGCAAAACAACCACTGATGACCAGTTGATTATGGAGAAAAATGGATCTAGATCGAGTCTGCCATCAATTCTAAAGCCATTGACACAGGATGTGGAAATTGTATGA

>protein

MSDRKMLFRQTTMFVKLFLVIVLYVDSSFGNNSSDITDLDCGPSEFACISSNKCVTLSWKCDGDPDCPDGEDEKNCSATTCTPNFFRCNNGLCISERWVCDGQDDCKDNSDEATEVCAERNTCVNKHKCSGTNMCIDPSLVCDGQPDCPEKDDEKDCSNLGCDKETEFKCHDNNGPCISSRWHCDGSDDCEDGSDEKNCTSQCEKGFKTCADGECINDLWWCDGDADCADKSDEAKCAELAVNQNCSETEFQCQGMSMVVQCVHMNWVCDGDSDCLDESDEKDCKPHTCNPNEIICDKNFCISELYVCDGDKDCEDGTDEFNCTVSCDVDSFDCGNQTCIPKTQLCDGFDHCIDGADESPLHCSSQVSSQTSCEHNNGGCMHLCKPNINSTGRTCECRPGYQLANHSNTDCQDINECETPGTCSQKCVNTKGSYKCDCEPGYTLIVPRYCKAIEGKHAVLILSDSHELRRYSLDEFRYSKLLENPVQRAVAMDFNIREHKVYWTDLQHKQVSVAGVNDVNHMKVIVNESIVKHPMKVIVNESIDMPDGLAVDWVHGNLYLSDTGLDKIEVLRLNGSHRKILVNTDLDEPRALVLDPQYGWIYWSDWGVQPKIEKCGMNGQNRKSIITKHIAWPNGLTIDYIQRRLYWVDAKLHTIGSSDLDGNNYKLILKNHGYLGHPFGITVFEDYLYWTDWMTNGVFKYSKFSQGNVTHVVMKLKTPMCIHAYHVVRQPSPMNHCGDNNGGCSHLCLPKPHEGVVAPTTPNYECACPDGMKFGEGEEKHHCVPSHEVVTNKIPTDQGKDMSNPNRLPYTIQTPPETQTTTKSSSSQETQTNFNQGETTDGVIPALNGTNSQLAHKESEGNGSVAIIVIVIVFVLGVTVAIIVALLIRRHKRKNVKSMNFDNPVYRKTTTDDQLIMEKNGSRSSLPSILKPLTQDVEIV


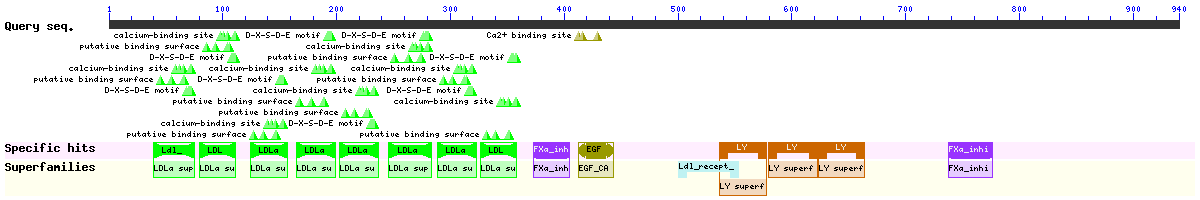


2) LDL-receptor 2_ NCBI number: PV615402

>mRNA

ATGAAAGCTGTGGCTTTCGCCATATCGCTTTTCGCTGCTCAACATGTTTTTTGCAGCCTAAAACTTGGCAGAGAATGCAATAGCTGCCCAAATAATTACTATATGTGTGGGGGAAAATGTGTCTGTGTTCCATCAAACTGGGTTTGTGATGGGGATGAAGACTGTGATGATGGCAGTGATGAGAAAAAAGAGGAATGCGACAATTACAAGTGCCCAGAGCTAGACTTTACTTGTGCCAATGGACACTGTATTCAGATGAAATGGGTCTGTGATGGAGATGATGATTGTGGTGACCATTCAGATGAGATCAGTTGTCCCCAGCGTAATTGCTCCAATGATGAACTCCACTGCAAGAATGGTAAATGTGTTCCCAAAGAATGGCGTTGTGATGGGGAAAATGACTGTGAGGATGAAACGGATGAAGACTGTCCTCTACAAAACTGTACAGCGGGTCAATTTCGTTGTCCGGATGGCTCCTGTATAGCACACTTATGGCAGTGTGATGGAGAAATAGATTGTATCAATGGAACAGATGAATCAGACTGTGGCCAAAAACAGCCCAAGTGCAGCAAAGGTGAGTTCCAGTGCAGTCGAACGTTCAGCTGTATCAGGCGAGAGTTTTTGTGTGATGATGAAAATGACTGCGGTGACTGGGAAGATGAAATAGACTGCGGTTATTGCCAGAAAGGAGAGTTTAACTGTACCAGTGGAGTCTGTATCAACAAAAGATGGAGATGTGATGGAGATTTTGACTGTGATGACAGGTCTGATGAGAGCAACTGCACTTTGCTGCCTTGTACATCAGAACAATTTCGTTGTGACAGTGGATTTTGTATTGATAAAAAACTGCAATGTGATGGTCTTAGAGACTGCGCTGATAACAGTGATGAAAAAAACTGCAAACCAAAGAGGTGCAAAGCTGAGCAGTTCCGTTGTGATGATGGGAAATGTTTAGGACTTCACAAAGTTTGTAATGGAAAGAATGAATGCCTTGATGGTTCTGATGAAATCAATTGTAATTCTGTGTCTCCTTGTTCTACTGATAATGGAGGATGCTCCCACTACTGCAAGGCTACCCCACAAGGAGCCCAATGCTGGTGTAAGCCTGGCTACAAGTTAGGGGATAACAAGACATTATGTAGAGATATTGATGAATGTCTTTTTGACGGGACCTGCAGCCAAATCTGCCGTAACACAGAAGGATCGTACAAATGTTCCTGTGTGACTGGATACCATTTGAAACCTGATGCCAGGGGGTGCAAGGCCCAAGGTGGGGAAGCTTATCTTATATTTGCCAATCGAGTGGACATAAGGAGAGTAACTCCTGACAAAAGTGAGTACACCTCCATACTCCAGGGACTCCAGAACGCTATAGCCCTGGACTTTCATCATGAGAAGGGGCTGGTCTTTTGGAGTGATGTGACACTGGACAAGATCAAAAGAGCCACTCTTAATGGAAGTGGTGTCATGGATATTGTTGCGGATGGTCTGGAAAATCCAGGTGGCATTGCCATCGACTGGATCCATGACAAACTATTCTGGACTGATGCAGGAACTTCAAGAATTGAGGTGTCAAACCTTGATGGTTCCTCAAGAAAAGTCATTTTGTGGCAAAATTTGGAGAAACCGAGAGCCATAGCAGCACATCCAAAGAAAGGTCTAATATTCTGGACTGACTGGGGAAACACGCCGAAAATTGAGAGGGCTGGAATGGATGGGAGTATGAGGATGATACTTGTAAACTCCTCTCTGTTCTGGCCCAATGGCATGACTGTTGATTATGCAGCAGAGAGGCTGTACTGGGCTGATGCCAAGCACCATGTCATTGAGTGTGCACATTTGGATGGGACCAACAGAAAGACTGTCATTAGTGAAGGTCTTCCTCACCCGTTTGCCTTGACCATCTTTGAGGATGAACTGTTCTGGACAGACTGGTACACGAAAAGCATAAACAAAGCCAACAAATTTAGTGGCAAGCGTGTTGAGACCATTCGGTCAAGCCTTTTCTTTCCAATGGATATCCATTCATTTCACCCCCAGCGCCAGCCACCTGCTGTTAATCTGTGTGGCTTGAACAATGGAGGATGCAGTCACCTGTGTCTGCCCAATGAGAGCGGCTACTCTTGCTCCTGTCCCACTGGACTTGTATTAAATAAAGACAGGAAAAACTGTGCTGAAGTCATGGATTTATTCATCCTATTTTCCACCCAAACGGATATACGTCGAATTTCATTTGATGTTGGGGAGTTGACGGATGTGGTTATACCACTAAGCGTAATTCAGAGTGCAGTTGGTGTGGAGTTTGACAGTGCTAAGGACACCATCTACTGGTCGGACATTGGAGCAGACCACATTGGAGAAGCATCATGGGATGGAAAAAATGAAAAGGTTATAATTGGTACAAGTTTAGATTCCCCCTCAGGAATAGCCTTTGACTGGGCTGGACGCAATCTGTATTGGACGGATAGCGGAAATGACCGTATAGAAGTTTGTGCCATTGACTCTAAACTGCGAACAGTTCTTATTTGGAGAGACCTTGACCATCCCAGAGATATTGTGGTCCACCCTAAACAAAGTTACATGTTCTGGACTGACCTGGGAAAGTCTGCCAAAATTGAAAGGTCTGGAATGGACGGCAGCGATCGGCAGGTTCTCATCAGCAACAACATGACGTGGCCGAATGGACTGGGCCTGGACTTTCAGAATGACCGCCTGTACTGGGTTGATGCTGGCACCCACACTCTGGAGTCATGCTCACTGGAAGGCAGTGACAGAAAGATCATTATTTCAGCAGGTCTCAAGCATCCGTTCGGTATAACCATCTATGACCACACCATGTACTGGACAGACTGGGACACAGGCAGCATACACTTTGCTGACAAAGACACCGGTGGCAGTCAAGGGGTGCTTGCCTCTGGCTTCGGTCAAATCATGGATCTCAAAGTGTTTCATCGAAACAGAATGCCAGTGTCAACGCCATGCAGCAGAAACAATGGCGGCTGTAGTCACATTTGTCTCCTGTCACCGCCACCTAAGGGTCACACCTGCGCCTGTCCGACCGGCATTATCCTTGGCAGTGATGGCAGACAGTGTCACTCAGAAATGAGAAATTTCCTCATCTTCACGAGACGTCAGGACATACGCAAAATTTCCCTGGAAGTTGAGTACTTCATGGACGTGGTCATTTCGGTAGGAGACCTCAGAAACGCCATAGCCATAGATGTTGATGTTTTAGAAGGAAAAATGTACTGGACAGACACAGTGCTGGACAGAATCTCCAGGGCCAACTTGAATGGATCTAATGTAGAGAAAGTTGTGGAGCATGGCATCCACACTGCAGATGGCTTGGCTGTGGATTCTGTTGGTAGAAAAATTTACTGGACTGACGATGGTCATAATCGGATACAGGTTGCTAATTTGGACGGCAGTATGAGGTCAGTGTTGTTATATGAAGACCTGGACAAGCCAAGGGCAATAGCACTGCATTATGACAAAGGCTACATGTTTTGGACCGACTGGGGGAAAAATGCCAGAATTGAGAGAGCTGATATGGATGGAAATAACAGACAAATCATCATCTCAGATGGAATAGCTTGGCCTAATGGTTTGACCATAGACAGACCAACAAACAGAATCATATGGGCTGATGCTCGAACAGAGTTAATTGAATGTGCTGATCTTTCTGGAAAATACCGTCGCAAGTTGGTCACCAAAGTTCGTCATCCTTATGGGCTAACAGTTGCAGGAAACTCCATTTACTGGACCGACTGGCGGGAAAGCTCGATACACCAAGCGAACAAAAACTTTGTGGCAAATATCACCCAAATAAGAGAAAACTTGCCTGGTATTATGGATATCCATGCGGTGCAGATTGATGGAATTCAAACACATGTTAATCGATGTGGAAAAAACAATGGGGGATGCAGCCATCTTTGTCTTCCCCATCCCAAAGGGATCTCATGTGCTTGTCCAACAGGGATCCTCATGAAGGCAGATGGAAAGACTTGTTACGATGCTCCCAGTAAGTACTTACTGTTTGCCGCACGGGGAAGCATTCGACGTATATCTTTGGACACACCTGACTTCACTGATGTGTACCTTCCTTTGCCTGACCTGCATAATGTCATTGCCCTGGACTTTGATTACCTTGAGAATATGGTATATTACACGGATGTGTATCTGGATGTTATTAGACGAGCATCTTTAAACGGAAGTCAGTGGATTGAAAATGTAGTGCTGAAGGAATTGGCCACAACTGATGGATTGGCTGTTGACTGGATTGCCAGGAATTTATATTGGACAGATTCTGGTCATGATGTAATCGAGGTGTCCCGTCTGGACGGTTCCAGCAGAAAAACAATTATTTCAGCAGATTTATTTGAGCCGAGAGCCATAACTCTGTTTCCCAGAAAAGGGTTGATGTTTTGGACCGACTGGGGAGATAAGCCAAAGATTGAGAGGTCCTACCTAGATGGAAGCACTCGAAAAGTCATTATCAACAGTCATTTAGGCTATCCTAATGCGCTGTCCATTGACTATGACACCCTGAGACTGTACTGGGTGGACGCCAAGCTTGATAAGATTGAGGCCTCCGACATGTCTGGGAAAAACAGGATGACCCTCATTCAACAGACTCCCCATCCATTTGGTCTGACTGTGTTTGAAGACTACATCTACTGGACTGACTGGCAAACTGAGAAGCTGGAGCGAGCCAACAAACTAGATGGCAGAAACAGGGTGACCGTACAGAGCAGGTTGGAGGGCCTGATGGATGTTCATTTGGTCTCTGCCATGAGACAGACAGGTTCCAATAACTGCAGTGTGAACAATGGAGGGTGCAGCCACCTTTGTCTGGCCAGACCTGATGGATATGTTTGTGCCTGCCCTAACAATGAAGATGCCAGGCCGTGTCAGACTGTTCCTTACCTAGGAAAACAAAGCTCTAACATTATCAACATTGACATCAATTCAGTACATGGCTGCTCAGATGAAGATAAGGCATTGGGTACATGTGTGGCAGAGGCAAGTTCAGACGGACAGAATCCAGCACCTTACATTGCTGTGGCTGTTACTCTCAGCATTTTGATCCTCATCATCATAACAGCATATTTTGTATGGAAGAGACAGAGAAGAAGACACTACAATGTAGAAGAGTTCTCTACCCTGACCTATGCCAACCCCACCTACCAGAAGGCCAGCACAGAAACTATCAACTCCGAAAACAGGAAAAACTATGCATGCTTTCGTTACCACGCCAGCGAAGAGCTCCTGACGACATGCCTAGGTGATGCCGACTGTGCAGCAAATCATAAAGAAAGTGTATCACTCATGCATCCCAACACTAACCTGTGA

>protein

MKAVAFAISLFAAQHVFCSLKLGRECNSCPNNYYMCGGKCVCVPSNWVCDGDEDCDDGSDEKKEECDNYKCPELDFTCANGHCIQMKWVCDGDDDCGDHSDEISCPQRNCSNDELHCKNGKCVPKEWRCDGENDCEDETDEDCPLQNCTAGQFRCPDGSCIAHLWQCDGEIDCINGTDESDCGQKQPKCSKGEFQCSRTFSCIRREFLCDDENDCGDWEDEIDCGYCQKGEFNCTSGVCINKRWRCDGDFDCDDRSDESNCTLLPCTSEQFRCDSGFCIDKKLQCDGLRDCADNSDEKNCKPKRCKAEQFRCDDGKCLGLHKVCNGKNECLDGSDEINCNSVSPCSTDNGGCSHYCKATPQGAQCWCKPGYKLGDNKTLCRDIDECLFDGTCSQICRNTEGSYKCSCVTGYHLKPDARGCKAQGGEAYLIFANRVDIRRVTPDKSEYTSILQGLQNAIALDFHHEKGLVFWSDVTLDKIKRATLNGSGVMDIVADGLENPGGIAIDWIHDKLFWTDAGTSRIEVSNLDGSSRKVILWQNLEKPRAIAAHPKKGLIFWTDWGNTPKIERAGMDGSMRMILVNSSLFWPNGMTVDYAAERLYWADAKHHVIECAHLDGTNRKTVISEGLPHPFALTIFEDELFWTDWYTKSINKANKFSGKRVETIRSSLFFPMDIHSFHPQRQPPAVNLCGLNNGGCSHLCLPNESGYSCSCPTGLVLNKDRKNCAEVMDLFILFSTQTDIRRISFDVGELTDVVIPLSVIQSAVGVEFDSAKDTIYWSDIGADHIGEASWDGKNEKVIIGTSLDSPSGIAFDWAGRNLYWTDSGNDRIEVCAIDSKLRTVLIWRDLDHPRDIVVHPKQSYMFWTDLGKSAKIERSGMDGSDRQVLISNNMTWPNGLGLDFQNDRLYWVDAGTHTLESCSLEGSDRKIIISAGLKHPFGITIYDHTMYWTDWDTGSIHFADKDTGGSQGVLASGFGQIMDLKVFHRNRMPVSTPCSRNNGGCSHICLLSPPPKGHTCACPTGIILGSDGRQCHSEMRNFLIFTRRQDIRKISLEVEYFMDVVISVGDLRNAIAIDVDVLEGKMYWTDTVLDRISRANLNGSNVEKVVEHGIHTADGLAVDSVGRKIYWTDDGHNRIQVANLDGSMRSVLLYEDLDKPRAIALHYDKGYMFWTDWGKNARIERADMDGNNRQIIISDGIAWPNGLTIDRPTNRIIWADARTELIECADLSGKYRRKLVTKVRHPYGLTVAGNSIYWTDWRESSIHQANKNFVANITQIRENLPGIMDIHAVQIDGIQTHVNRCGKNNGGCSHLCLPHPKGISCACPTGILMKADGKTCYDAPSKYLLFAARGSIRRISLDTPDFTDVYLPLPDLHNVIALDFDYLENMVYYTDVYLDVIRRASLNGSQWIENVVLKELATTDGLAVDWIARNLYWTDSGHDVIEVSRLDGSSRKTIISADLFEPRAITLFPRKGLMFWTDWGDKPKIERSYLDGSTRKVIINSHLGYPNALSIDYDTLRLYWVDAKLDKIEASDMSGKNRMTLIQQTPHPFGLTVFEDYIYWTDWQTEKLERANKLDGRNRVTVQSRLEGLMDVHLVSAMRQTGSNNCSVNNGGCSHLCLARPDGYVCACPNNEDARPCQTVPYLGKQSSNIINIDINSVHGCSDEDKALGTCVAEASSDGQNPAPYIAVAVTLSILILIIITAYFVWKRQRRRHYNVEEFSTLTYANPTYQKASTETINSENRKNYACFRYHASEELLTTCLGDADCAANHKESVSLMHPNTNL


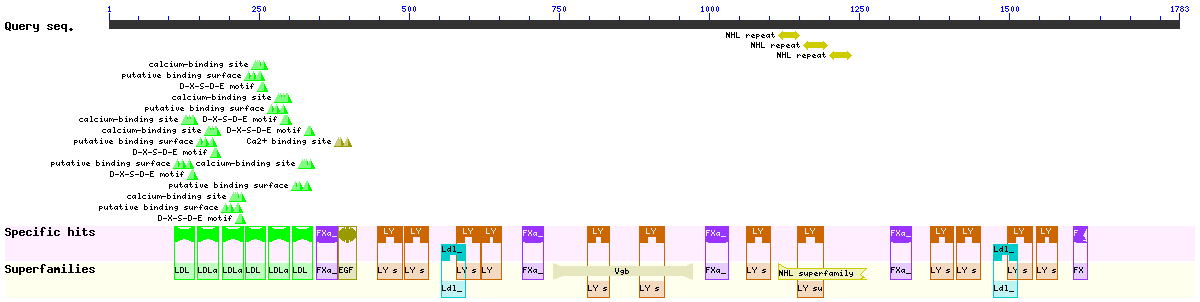


3) Sterol carrier protein 2_ NCBI number: PV615403

>mRNA

ATGGACCAAAGACGAATATTTGTTATAGGTGTCGGAATGACTAAGTTTGAAAAACCAGGCGGAAGAAATGACTTTGATTATCCTCAAATGGCTCTAGAAGCTGGCACTAAAGCTCTAGAAGATGCAGGGATTAATTATGATCAAATTGAGCAAGCTTGTGTTGGTTATGTATATGGAGACACCTGCTGTGGGCAAAGAGCTCTGTATCAACTTGGTCTGAAAGGCATTCCGATATACAATGTAAACAATGCTTGTGCCACTGGTTCAACTGCACTTTTTATGGCAAAAAATATGATTGCTGGTGGCTTAGCAGACTGTGTCATGGCAATAGGATTTGAAAAAATGGAGAGAGGTTCTTTAAAACCAAAATGGACAGATCGAACAAATCCAATGGACAAACATATGGAGGTAATGATAGAAACTCGTGGATTCGATAAATCTCCAGCAACAGCACAGATTTTTGGAAATGCAGGAAGGGAACACATGGAGAAATATGGTACAAAGCCAGAACATTTTGCTAAAATTGCCTGGAAAAATCACTTGCACTCAACAAACAACCCCTATTCACAGTTCCAGACAAAATACAGCTTGGCAGAAATCCAGAACTCTCCAATGGTTTACTTTCCTCTAACAAAACTTCAATGCTGCCCCACATCAGATGGATCAGCTTGTGCTATTCTAGCTAGTGAGGATTTTGTAGTTAGAAATGGATTACAGGGCCAGGCAGTGGAAATATTGTCTATGGAAATGGGCACTGACCAGGCTAGTGTGTTTAATGAAGGCAGCTGCATAAAAATGATTGGTTACGATATGGTTGAGAAGACTGCTCAAAAGACATTTCAAAAAGCAGGATTAAAACCTTCAGATGTCCAAGTTGTAGAGCTGCATGACTGCTTCTCTTGCAATGAATTGTTAACTTATGAAGCACTTGGCTTGTGTTCTCAGGGTGAAGCAGGTCTCTTTGTGGATAGAGGTGACAATACATATGGTGGAAAGTATGTGGTCAATCCTAGTGGTGGATTAATTTCTAAAGGACATCCACTTGGTGCCACAGGTTTGGCACAGTGTGCAGAATTGTGCTGGCAGCTTAGAGGAATGGCAGGAAAAAGACAAGTACCAAATGCTAAAGTTGCTTTACAGCACAATATTGGACTTGGTGGAGCTGCTGTTGTTTCCTTGTATAAACTTGGCTTTCCACAATCTTTATCTCGAGATATTCAACTAGCTGCAGTTAATGCATCAACTGAGAAAGATTTTAAATCGGCAGCACTGTTTTCAGAAATTAAAAAAAGATTAGAACAGGATGGAGTTAATATTGTTAAAAAGATGAAAGGTGTCTTCTGTTTCAAGGTAAAGGGACAAGGAGACAAGGAAGCCGTCTGGATTGTTGATGTGAAAAATGGAAATGGTTCTGTTAAATTTGATTCAAATGATAAGGCAGATACAACTATTGTCATGTCAGATCAAGATTTGTGTGACTTAATGATGGGCAAACTCAACCCTCAAACTGCATTTTTTCAAGGCAAGCTCAAGATAACTGGCAACATGGGGTTAGCAATGAAATTGAAAGAATTGCAACCCAAATCTGATGCAAAGCTCTAA

>protein

MDQRRIFVIGVGMTKFEKPGGRNDFDYPQMALEAGTKALEDAGINYDQIEQACVGYVYGDTCCGQRALYQLGLKGIPIYNVNNACATGSTALFMAKNMIAGGLADCVMAIGFEKMERGSLKPKWTDRTNPMDKHMEVMIETRGFDKSPATAQIFGNAGREHMEKYGTKPEHFAKIAWKNHLHSTNNPYSQFQTKYSLAEIQNSPMVYFPLTKLQCCPTSDGSACAILASEDFVVRNGLQGQAVEILSMEMGTDQASVFNEGSCIKMIGYDMVEKTAQKTFQKAGLKPSDVQVVELHDCFSCNELLTYEALGLCSQGEAGLFVDRGDNTYGGKYVVNPSGGLISKGHPLGATGLAQCAELCWQLRGMAGKRQVPNAKVALQHNIGLGGAAVVSLYKLGFPQSLSRDIQLAAVNASTEKDFKSAALFSEIKKRLEQDGVNIVKKMKGVFCFKVKGQGDKEAVWIVDVKNGNGSVKFDSNDKADTTIVMSDQDLCDLMMGKLNPQTAFFQGKLKITGNMGLAMKLKELQPKSDAKL


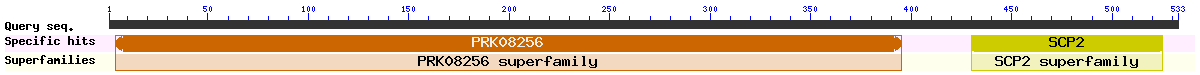


4) Scavenger receptor class B type 1_ NCBI number: PV615404

>mRNA

ATGGCATCCAAGAAGTCTGTGGGGCTGATTTTGTGTGGTCTGTTGGGCGTGGCATTTCTAGTCACTGGCTGTGTTCTCATTCATGTGTTTGATGAACTTGCACATTCAGAGATTGATGATAATCTTCCACTGAAACCCGGCTCAGACTCCTACAAAAACTGGCAGTCTCCCAAGACACCCATTTACTTCCAAGTTTGGTTCTTTGATATAGTCAATCCCCTAGAAGTTCTGGCGGGGGAGAAGCCAGCTGTTATACAGAAAGGACCTTACACTTACAGGGAGCACCGTGAGAAGTTTAATATCACCTACTATGACAACGGCACACTGACGTACTTGGAGAACCGCTCATTTAATTTTGACAGGGAGATGTCTGTGGGCTCAGAGAATGACACCTTCAACACCATAAGTCTGCCTGGTCTGACAATTATGGAGATGTTGAAGTGGGAACTCCAGGCCATCAAGTGGTTTGTCAATCTTATCCTTAAAGAGACAGGCGAGAACTTGATACAAGAGCTCTCAGTCCATGACCTGATGTGGGGCTATGAGGATCCCTTGCTGAAGGCAGCCAAGAAAGCAGCAGCGGAATTCAAAATACATCTGAACTTCTCAGATATGTTTGGTTTGTTTTATAATCAAAACAACTCCAATGATGGCCTGTACCAGATATACTCGGGGATGAAAGATCTTGACAACTTTGGGAATATTGTTTCCTGGAATGGTCAAACGGAGCTAAATTATTGGACCAATGACTCCGCCAACATGATCAATGGAACAGATGGGACCATTTACCCACCATTCATCGACTTGAAGGCCACAAAATATCTCTTCTCCTCTGATCTCTGTAGGTCTTTGGGCATGACCTATACCAAAGGTGTGAGTGTCAAGAGCATTGACCTGGCCAGGTTTGTTGCACCTGACATCATGTTTGGGAATGTATCGACCAACCCCTACAACGCTGGCTTCTGCACACCCTCCGGCAACTGCCTCCCATCTGGGCTCCTCAACGTCAGTGTCTGTCGGACTGGGGCTCCCGTCATAATGTCAATGCCGCACTTCCTCGGGTGCGACCCGGAGACGGTCAATGCCATCAGGGGCCTGAGACCCAACAGGGACGAACATCAGAGTTACATTGACATAGAGCCCATGACTGGTGTTGCCATGAGTGTGGGTAAAAGGCTTCAGATCAACACCTACTTGGAATTTATTGAGGGCTTTGATGATCTGAAGAACATCAAAAAGCCTATTTTCATGCCAGTCATGTGGTTGAATGAAAGCGCCCTGATATCTGACCAAGATGCTGGTGATTTCAAATCTCAAGTGGTTGACAAAATAACCCTCACGAAAGCTGTCAAGTTTGGCCTCATTGCCCTGGGTGCTGTCATGATCCTTTGTGTGATTCTAGTGCTGGTGGCAACCAAACTCAAAACGTGCCACAATGCAAAAGTTGTTGATGATGAGAATGAACCAATACTGCATCCTCACAATGCTGCAGTGTCGACATGA

>protein

MASKKSVGLILCGLLGVAFLVTGCVLIHVFDELAHSEIDDNLPLKPGSDSYKNWQSPKTPIYFQVWFFDIVNPLEVLAGEKPAVIQKGPYTYREHREKFNITYYDNGTLTYLENRSFNFDREMSVGSENDTFNTISLPGLTIMEMLKWELQAIKWFVNLILKETGENLIQELSVHDLMWGYEDPLLKAAKKAAAEFKIHLNFSDMFGLFYNQNNSNDGLYQIYSGMKDLDNFGNIVSWNGQTELNYWTNDSANMINGTDGTIYPPFIDLKATKYLFSSDLCRSLGMTYTKGVSVKSIDLARFVAPDIMFGNVSTNPYNAGFCTPSGNCLPSGLLNVSVCRTGAPVIMSMPHFLGCDPETVNAIRGLRPNRDEHQSYIDIEPMTGVAMSVGKRLQINTYLEFIEGFDDLKNIKKPIFMPVMWLNESALISDQDAGDFKSQVVDKITLTKAVKFGLIALGAVMILCVILVLVATKLKTCHNAKVVDDENEPILHPHNAAVST


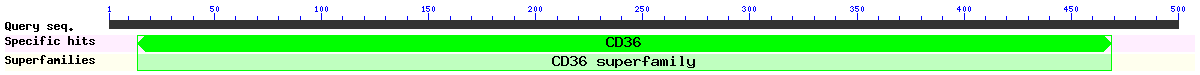


5) STARD1/D3 homolog 1_ NCBI number: PV615405

>mRNA

ATGGCGCACATCAGTAAGATCTACACCATGGGACCCAATCACCACCCGAGCGTCACTTTTGTCCGGCAGATCAGCGATGTAGCCGTGGTGGAGTCAGACCGGGACAACCAGGCCAAGCAGGCCGGCCATCTCATCTACAGCAGCATACATGGTGACGGCAGCAGCAGCTCGTTGATACCAGCTCTGCAATCTTCTACTGACTCCTACTCTGGTCCGATGCCCCCTCCGGCCATCAATCCAGCTCTCAACAGCAGCATGGAGAGAGCCGGCTCCCTGGGTCAGGGCCGGATGTCATCCGTGAGGCGGACATTTTGTCTGCTGGTTTTGTTTGATCTTATTCTCATGTTTATCCTGTGGGTCATCTACACTCAGCTGATCGGTGACACGGGTTTCAAAGCTTTTGATAAGCAAGTCATCAATTACGATTTCAAGACATCTCTCTTTGATTCGGTGATGCTGTCAGCGTCTAGATTCACACTTCTGCTGTTGGCCTATGCTTTGTTTAGAATCCAGCACTGGTGGATGATTGCTCTCTGCACTGCTGCCTCCTGTGCCGTTCTCATCGCCAAGATCTTCATTTTTGATTTTGAAGGCACCAAGAGCAGCAACAACCCCCTGAGTTACTGCCTCATCATTATATCCTTTGTGCTGGCTTGGGCCGAGACCTGGTTCCTGGACTTCAAAGTTTTACCGCAGGAGCAAAAGGCTCTTGAAAGACTCCGCCTTCACAGCACCCCCAACTATGGCTCGATCTATGTCCCCATCAGGCCCAGGTCCATGGTCGGAGATGACATGCAGTCCATTATCACAGAGGACAACAATCAGTTTTACTCACCTGTTGAGTCACCAGCCGAGTCAGACACAGAGGAGGGAACTCATGCACAGGGAGTTGGGGACAGGACCAGCGTGTACCGCAGTGTTCCCGTGTCAAAGGTCACAACACGCCAGGCAAGCCAAGCATCTATTAACACACTAGCCTTAGAGGAGAATGATTACATCCGGCTGGCCAAGCATTCCTGGGAGGTCCTGTGGACATACTACACCAGTCCAGAATCTGACTGGAAGCTGGAGACGGGGAACAATGAGATGACGGGGGTCGTTCACAGCAAAAAGGTCAAGAGTGTCGGAAAGGTGTTCCGATTAAGGGGTATTGTAGACATGCCAGCCAAAGAGCTGTATGAGGAGATGACATTCAAACCAGAACTGCAGTCCGCATGGAATAAAGCCATCAAAGAATCTAGGGTTTTACAAGTTGTGGATGATCACACGGACGTCCTCTACAACGTTGCTGCGGAGATCGCCGGCGGTGTGATCACCAGTCGAGACTTTGTCAGTTTACGCACCTGGGGTCAACGGGACGGCGTTTACATTGGTTCTGGTATGGGCGTCAATCATCCTGACATGCCACCGCAGAAAAATTATGTCAGAGGCACAAATGGAGCTGGTGGCTGGGTGTATAGACCTTGCCCGGGAGACGCTAGTAAAACTTTATTTTTCTGGTTCATGAATACAGACATCAAGGGCTGGTTTCCTCAGTCCCTGATTGACGCCAACATGGCCAAAGTCCTCATGGATTTCATTCAAGACCTGCGCGTCCATGTCAAAAGTATAACACACTCCTAG

>protein

MAHISKIYTMGPNHHPSVTFVRQISDVAVVESDRDNQAKQAGHLIYSSIHGDGSSSSLIPALQSSTDSYS

GPMPPPAINPALNSSMERAGSLGQGRMSSVRRTFCLLVLFDLILMFILWVIYTQLIGDTGFKAFDKQVIN

YDFKTSLFDSVMLSASRFTLLLLAYALFRIQHWWMIALCTAASCAVLIAKIFIFDFEGTKSSNNPLSYCL

IIISFVLAWAETWFLDFKVLPQEQKALERLRLHSTPNYGSIYVPIRPRSMVGDDMQSIITEDNNQFYSPV

ESPAESDTEEGTHAQGVGDRTSVYRSVPVSKVTTRQASQASINTLALEENDYIRLAKHSWEVLWTYYTSP

ESDWKLETGNNEMTGVVHSKKVKSVGKVFRLRGIVDMPAKELYEEMTFKPELQSAWNKAIKESRVLQVVD

DHTDVLYNVAAEIAGGVITSRDFVSLRTWGQRDGVYIGSGMGVNHPDMPPQKNYVRGTNGAGGWVYRPCP

GDASKTLFFWFMNTDIKGWFPQSLIDANMAKVLMDFIQDLRVHVKSITHS


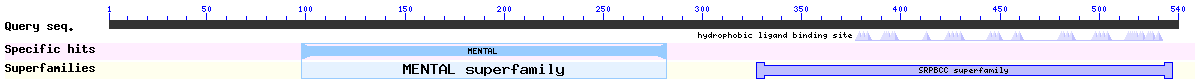


6) STARD1/D3 homolog 2_ NCBI number: PV615406

>mRNA

ATGTCTTCAACTATTATGTCCATTAATACGGATGTAGCCGTGGTGGAGTCAGACCGGGACAACCAGGCCAAGCAGGCCGGCCATCTCATCTACAGCAGCATACATGGTGACGGCAGCAGCAGCTCGTTGATACCAGCTCTGCAATCTTCTACTGACTCCTACTCTGGTCCGATGCCCCCTCCGGTCATCAATCCAGCTCTCAACAGCAGCATGGAGAGAGCCGGCTCCCTGGGTCAGGGCCGGATGTCATCCGTGAGGCGGACATTTTGTCTGCTGGTTTTGTTTGATCTTATTCTCATGTTTATCCTGTGGGTCATCTACACTCAGCTGATCGGTGACACGGGTTTCAAAGCTTTTGATAAGCAAGTCATCAATTACGATTTCAAGACATCTCTCTTTGATTCGGTGATGCTGTCAGCGTCTAGATTCACACTTCTGCTGTTGGCCTATGCTTTGTTTAGAATCCAGCACTGGTGGATGATTGCTCTCTGCACTGCTGCCTCCTGTGCCGTTCTCATCGCCAAGATCTTCATTTTTGATTTTGAAGGCACCAAGAGCAGCAACAACCCCCTGAGTTACTGCCTCATCATTATATCCTTTGTGCTGGCTTGGGCCGAGACCTGGTTCCTGGACTTCAAAGTTTTACCGCAGGAGCAAAAGGCTCTTGAAAGACTCCGCCTTCACAGCACCCCCAACTATGGCTCGATCTATGTCCCCATCAGGCCCAGGTCCATGGTCGGAGATGACATGCAGTCCATTATCACAGAGGACAACAATCAGTTTTACTCACCTGTTGAGTCACCAGCCGAGTCAGACACAGAGGAGGGAACTCATGCACAGGGAGTTGGGGACAGGACCAGCGTGTACCGCAGTGTTCCCGTGTCAAAGGTCACAACACGCCAGGAGAATGATTACATCCGGCTGGCCAAGCATTCCTGGGAGGTCCTGTGGACATACTACACCAGTCCAGAATCTGACTGGAAGCTGGAGACGGGGAACAATGAGATGACGGGGGTCGTTCACAGCAAGAAGGTCAAGAGTGTCGGAAAGGTGTTCCGATTAAGGGGTATTGTAGACATGCCAGCCAAAGAGCTGTATGAGGAGATGACATTCAAACCAGAACTGCAGTCCGCATGGAATAAAGCCATCAAAGAATCTAGGGTTTTACAAGTTGTGGATGATCACACGGACGTCCTCTACAACGTTGCTGCGGAGATCGCCGGTGGTGTGATCACCAGTCGAGACTTTGTCAGTTTACGCACCTGGGGTCAACGGGACGGCGTTTACATTGGTTCTGGTATGGGCGTCAATCATCCTGACATGCCACCGCAGAAAAATTATGTCAGAGGCACAAATGGAGCTGGTGGCTGGGTGTATAGACCTTGCCCGGGAGACGCTAGTAAAACTCTATTTTTCTGGTTCATGAATACAGACATCAAGGGCTGGTTTCCTCAGTCCCTGATTGACGCCAACATGGCCAAAGTCCTCATGGATTTCATTCAAGACCTGCGCGTCCATGTCAAAAGTATAACACACTCCTAG

>protein

MSSTIMSINTDVAVVESDRDNQAKQAGHLIYSSIHGDGSSSSLIPALQSSTDSYSGPMPPPVINPALNSSMERAGSLGQGRMSSVRRTFCLLVLFDLILMFILWVIYTQLIGDTGFKAFDKQVINYDFKTSLFDSVMLSASRFTLLLLAYALFRIQHWWMIALCTAASCAVLIAKIFIFDFEGTKSSNNPLSYCLIIISFVLAWAETWFLDFKVLPQEQKALERLRLHSTPNYGSIYVPIRPRSMVGDDMQSIITEDNNQFYSPVESPAESDTEEGTHAQGVGDRTSVYRSVPVSKVTTRQENDYIRLAKHSWEVLWTYYTSPESDWKLETGNNEMTGVVHSKKVKSVGKVFRLRGIVDMPAKELYEEMTFKPELQSAWNKAIKESRVLQVVDDHTDVLYNVAAEIAGGVITSRDFVSLRTWGQRDGVYIGSGMGVNHPDMPPQKNYVRGTNGAGGWVYRPCPGDASKTLFFWFMNTDIKGWFPQSLIDANMAKVLMDFIQDLRVHVKSITHS
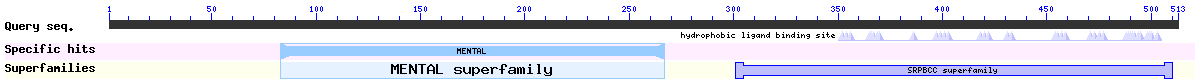


7) STARD1/D3 homolog 3_NCBI number: PV615407

>mRNA

ATGAGTTTTAACGAAGACGTTTGTTGTTTCTGTCACCAGGAGAATGATTACATCCGGCTGGCCAAGCATTCCTGGGAGGTCCTGTGGACATACTACACCAGTCCAGAATCTGACTGGAAGCTGGAGACGGGGAACAATGAGATGACGGGGGTCGTTCACAGCAAAAAGGTCAAGAGTGTCGGAAAGGTGTTCCGATTAAGGGGTATTGTAGACATGCCAGCCAAAGAGCTGTATGAGGAGATGACATTCAAACCAGAACTGCAGTCCGCATGGAATAAAGCCATCAAAGAATCTAGGGTTTTACAAGTTGTGGATGATCACACGGACGTCCTCTACAACGTTGCTGCGGAGATCGCCGGCGGTGTGATCACCAGTCGAGACTTTGTCAGTTTACGCACCTGGGGTCAACGGGACGGCGTTTACATTGGTTCTGGTATGGGCGTCAATCATCCTGACATGCCACCGCAGAAAAATTATGTCAGAGGCACAAATGGAGCTGGTGGCTGGGTGTATAGACCTTGCCCGGGAGACGCTAGTAAAACTTTATTTTTCTGGTTCATGAATACAGACATCAAGGGCTGGTTTCCTCAGTCCCTGATTGACGCCAACATGGCCAAAGTCCTCATGGATTTCATTCAAGACCTGCGCGTCCATGTCAAAAGTATAACACACTCCTAG

>protein

MSFNEDVCCFCHQENDYIRLAKHSWEVLWTYYTSPESDWKLETGNNEMTGVVHSKKVKSVGKVFRLRGIVDMPAKELYEEMTFKPELQSAWNKAIKESRVLQVVDDHTDVLYNVAAEIAGGVITSRDFVSLRTWGQRDGVYIGSGMGVNHPDMPPQKNYVRGTNGAGGWVYRPCPGDASKTLFFWFMNTDIKGWFPQSLIDANMAKVLMDFIQDLRVHVKSITHS


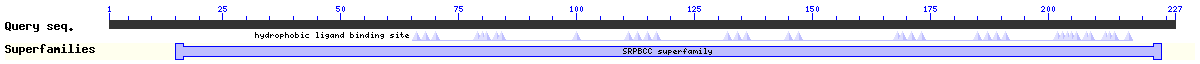


8) Niemann-Pick intracellular cholesterol transporter 1_ NCBI number: PV615408

>mRNA

ATGGGAGGATCTCATTTGATGGGCTGGCGCTTCCTTGTATGTGTTATCGTGGGACTTAATATTATTTCATTGTGCAATGGTGACTGTATATGGTATGGACAGTGCGGGTACAACACGGGAATAGCCAAAGACAAGAAGCTCAACTGTAAATATAGTGGCCCGCCTCAGGCTTTTCAGACAGAAGAAGGGGAGAGACTCTTTGCTGAGAATTGCCCAAATCTCTACTCTGGGCCTGGGGTGAAGACATGTTGCTCCGACGATCAGATAATGACACTGAACACACAGATTGCTGTTCCCAGGCAGGCGTTGCAACGTTGCCCGAGCTGCCTGCACAACTTCTTGAGTGTCTGGTGCTATTTAACGTGCGGAGATAAGCAGAGTGAGTTTATGTCGGTGAATTCCACAATACCTTACAATGTCACCCAAACGGCCATTGTTTCTGTGGAGTACGCCATTCGCACAGACTCGGCACAAGGGATGTTCAACTCGTGCCGTAACGTGCAGATGCCGAGCACCAACGAGCCAGCTCTGTCCATTCTGTGCGGTGAGGACGCTGACACTTGCACTGCTAAGAAGTGGCTGGATTACATGGGGACAATTCAGAATGGCCAGGCACCCTTCGACATCCACTTCCATCTGGATAATAAGAACATTACTATAAATGGCACAAATCTCTCACCTTTGGATATTCAACCCGTCCCATGCAGTAAAGCCCCCTCAAACACCACCGAGCCGTGCAGTTGCCAGGACTGCACAGCGTCCTGCACCCCGACGCCCCCGCCGTCCCCGCCCGAGGCTCCCTGTGAGATCCTGCACATTGACTGCTACTACTTTATCTTCGGGGTCATCTACGCGGTCTTTGTCCTGGTGTTCGGCTGCTACTCCATCTGTTACAACATAATAGTTCAGGACTCCCTCCGCCTGGAGGAGCCGGGAGAGGAAGACAGCCCAGGGTGTTGTGGGACAAGAGGTTGGGCCGTGACAAGGAATGCACGCAAGCAGCAGTACCTGGAAAACATCAGCAAAGACCAGCTGGGTCGCTGTGAAAACATTGGAGCCAAAATGGAGGCCTTTCTGGAAAGGTCATTCCGACGATGGGGCTATTTCTGTGCCATCCATCCATTCCTTGTTTCTATCATTGTTTTGGCGGTATTTGGGGGCCTTTGTGCTGGTGCTGCATTCTTCACAGTGACCACTGATCCTGTCAAATTGTGGTCATCCCCTGACAGTCGGGCTCGGACAGAGAAAGACTACTTTGACTCCCATTTTGTTCCATTTTACCGCACAGAACAGCTGATCATCAGCAGGCCTGAAAACACAACCGTCGTCCAATGGAAGAATCTGAATTACACCAACCTGTTTGATAAAACATTCTTACATGAGATTCTGGATCTGCAGTTGGCTATAGCAGAACTCCATGCAGAGTACCTGGGTAAGAATGTGAGCCTGACTGACATCTGCTTTGCGCCCATGAGTCCCGAGAACGACAACTGCACCATCCAGTCTGTGCTCAACTACTACCAGAACTCTCACGACATGCTGGACCTGGAGGCCCTGGATGACTACGGCTGGTTCGTTGTGGCCAACTACCTTGATCACTTTGAGTATTGTGTTCAATCTCCCTTCTCTCTGAATGACACAACCAAACTACACACACCGTGTCTGGGAACGTACGGAGGACCTGTCTTTCCATGGGTGGCCCTCGGAGGGTATGAAGGCAAATCCTACAAAACGGCCAATGCGCTGGTCATCACCATTCTAGTCAACAATCATTTGGATGATAAACTCAATGGTCCGGCCAAGGCCTGGGAGACCAAGTACATAGAGTTCATGAAGAACTACACAGCTGATCATCCAGACATCCACATTGCTTTCTCCTCTGAGAGATCTATAGAAGACGAGATTGATCGGGAAAGTGAGAGCGACATTCTCACCATACTGCTGAGTTACCTCATCATGTTTGGCTACATCACCATCACTCTGGGGCAGTACACAGACCTGTCAAGGATTGTGGTCGATGCCAAAGTTTCACTGGGTCTGTCTGGCGTGTTGATTGTGCTGCTGTCTGTGGGTGGATCTCTGGGTTTCTACAGCTACATCGGCATCCCATCCACACTCATCATTGTGGAGGTGGTTCCCTTCTTGGTGCTGGCTGTCGGTGTGGATAACATTTTCATCCTTGTGCAGACCTGCCAGAGGGAGCCCTTGCATGAAGGAGAGACTGTTGAACACAAAATCGGTCGTGTGGTTGGTAAAGTTGGACCAAGTATGTTGCTGACAAGTTTGTCCGAGTCTCTGGCATTCTTCCTAGGAGCCCTCACACAGATGCCGGCAGTGAAGGTGTTTTCATTGTATGCCGCCATGGCAGTGTTGTTTGATTTCATTCTGCAAATCACCATTTTTATATCACTCTTGACAGTGGACGCCAAACGCCAGGAGTCAAACAGAATTGATGTCCTCTGCTGCTACAAGGTACCCCTAAAAAAGTCCAAGCCAAGAAATGGCCTCCTGTACACATTTGTCAAGGAATACTACTCCCACTTTTTAATGAAGGAATGGGTCAGGCCCATTGTTATGCTGACGTTTGTTGGCTGGTTCTGTGCTAGTGCCGCCATGACCTCTAAAGTTGAGATTGGTTTGGATCAAGCCCTGTCAATGCCTAAGGATTCCTACGTTTTGAATTACTTCGGTAACCTGACCAGATACCTCTCTGTCGGAGCCCCTGTCTACTTTGTGATGGAGTCCGGCCATGACTTCTCCACATATTCTGGTCAGAACGATGTATGTGGTATAAGTGGCTGTCCGCAGAACTCACTTGTCCAGCAGGTCAACCACGCTGCCTTGAACCCAAACTACACTTATGTGGCTCAGCCAGTCTCCTCTTGGCTGGATGATTACTTCTCATGGCTGTCCAGCCCTGCGTGTTGCAGGTATAAAAATGACACCGGGGAGTTCTGCCCTTCTACAGACTCCAACTCTAGTTGCATCCCATGCCCACTAGACCACTTAGAAAAAGGCCGTCCCGTTGGAGAGACCTTCAACAGATATTTGAAATGGTTTCTGTCAGACAACCCTGGATTAAAGTGTGGCAAAGGTGGCCATGCGGCCTACGGTTCCGGTGTTCAGCTCTTCAAGGATCCAAACAATGAGACACATGTTGGAGCTTCCTACTTCATGACCTACCACAACATCCTCAAGACATCGGCTGACTACATCAACGCTCTGAAAGAAGCCAGGGTGATTGCTGACAACATAACCAGCACACTTCAGCATAGCGGGCGTCAAGGCAAGGTGTTTCCCTACAGTGTGTTCTATGTGTTCTACGAGCAGTACCTGACCATCATCAAAGACTCCATCTTGAACATGGTCTACTGTCTGGCTGCCATTCTGGTCATCACATTCGTCCTGCTGGGCTTTGACCTGTACTCGTCTGTTGTGGTGGTGCTGACCATCATCATGATTCTCATTGACATCATGGGGATGATGTTCCTGTGGGGGATCGATCTCAATGCACTGTCCTTGGTCAATCTCATCATGGCCATTGGAATATCCGTCGAGTTTTGCTCACACATCATCCGAGCTTTTGCCATCAGCGTCCTACCAACTCGCAAGGAGAGAGCCAAAGAGGCCCTGGCTCACATGGGAAGCTCGGTCCTGAGTGGTATCACCCTCACCAAGCTGGGTGGTATCATCATGCTGGGCTTCTCCAAGTCCCAGCTGTTCCAGGTGTTCTACTTCAGGATGTACCTGGGCATTGTCGTGTTTGGGGCCACCCACGGCCTCATCTTCCTACCTGTGTTCCTCAGCTACCTGGGTCCCCCCGTCAACAAGGCCAAAGTTTACTACAGTAAACAGGAGGACAGCACAGACGAGACGGACGACACGGGCTCTGCCCGTCAACCCAACGGAGACGCCACGCACAGATCGGCGCTGAATGGTGGCGCCACATACAGACAAACTCACGACAACCCCCCAGCCTACAACACTATAAGCAGAGCCTCAACATAA

>protein

MGGSHLMGWRFLVCVIVGLNIISLCNGDCIWYGQCGYNTGIAKDKKLNCKYSGPPQAFQTEEGERLFAENCPNLYSGPGVKTCCSDDQIMTLNTQIAVPRQALQRCPSCLHNFLSVWCYLTCGDKQSEFMSVNSTIPYNVTQTAIVSVEYAIRTDSAQGMFNSCRNVQMPSTNEPALSILCGEDADTCTAKKWLDYMGTIQNGQAPFDIHFHLDNKNITINGTNLSPLDIQPVPCSKAPSNTTEPCSCQDCTASCTPTPPPSPPEAPCEILHIDCYYFIFGVIYAVFVLVFGCYSICYNIIVQDSLRLEEPGEEDSPGCCGTRGWAVTRNARKQQYLENISKDQLGRCENIGAKMEAFLERSFRRWGYFCAIHPFLVSIIVLAVFGGLCAGAAFFTVTTDPVKLWSSPDSRARTEKDYFDSHFVPFYRTEQLIISRPENTTVVQWKNLNYTNLFDKTFLHEILDLQLAIAELHAEYLGKNVSLTDICFAPMSPENDNCTIQSVLNYYQNSHDMLDLEALDDYGWFVVANYLDHFEYCVQSPFSLNDTTKLHTPCLGTYGGPVFPWVALGGYEGKSYKTANALVITILVNNHLDDKLNGPAKAWETKYIEFMKNYTADHPDIHIAFSSERSIEDEIDRESESDILTILLSYLIMFGYITITLGQYTDLSRIVVDAKVSLGLSGVLIVLLSVGGSLGFYSYIGIPSTLIIVEVVPFLVLAVGVDNIFILVQTCQREPLHEGETVEHKIGRVVGKVGPSMLLTSLSESLAFFLGALTQMPAVKVFSLYAAMAVLFDFILQITIFISLLTVDAKRQESNRIDVLCCYKVPLKKSKPRNGLLYTFVKEYYSHFLMKEWVRPIVMLTFVGWFCASAAMTSKVEIGLDQALSMPKDSYVLNYFGNLTRYLSVGAPVYFVMESGHDFSTYSGQNDVCGISGCPQNSLVQQVNHAALNPNYTYVAQPVSSWLDDYFSWLSSPACCRYKNDTGEFCPSTDSNSSCIPCPLDHLEKGRPVGETFNRYLKWFLSDNPGLKCGKGGHAAYGSGVQLFKDPNNETHVGASYFMTYHNILKTSADYINALKEARVIADNITSTLQHSGRQGKVFPYSVFYVFYEQYLTIIKDSILNMVYCLAAILVITFVLLGFDLYSSVVVVLTIIMILIDIMGMMFLWGIDLNALSLVNLIMAIGISVEFCSHIIRAFAISVLPTRKERAKEALAHMGSSVLSGITLTKLGGIIMLGFSKSQLFQVFYFRMYLGIVVFGATHGLIFLPVFLSYLGPPVNKAKVYYSKQEDSTDETDDTGSARQPNGDATHRSALNGGATYRQTHDNPPAYNTISRAST
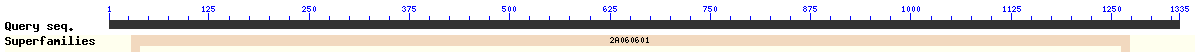


9) Niemann-Pick intracellular cholesterol transporter 2_NCBI number: PV615409

>mRNA

ATGGAAAGTTTCGTGTGGTTTGTGACACTTCTTGCTCTGGCGAGTGCGGAGAAAATCGCGTTCAAGCCTTGTCCTGGAGACAACCTTGGACAAGCAACCTCCGTTGAACTGAGCCCTTGCCCCAGCCAGCCTTGCACATTCAGCCATGGCAGCACAGTCACAGTGGTCATCGAGTTCACTGCAGTTAACGACTCCACCGTCCTGGACTCTAGAGTGTATGGCATCGTAGAAACTGTCCCCGTGGAGTTCCCGCTGCCCAATGGAGATGGATGCAAGGACAGCGGCATTGCCTGCCCTCTGGTCAAGGGTCAAAGCTACAAGTATACGTCATCTTTTCCAGTTCTGAACACCTATCCTGTGATCTCCCTTGTGGTGATGTGGAAGCTGCAAGCAATTTCTGGTAATCTCGTCTGCTTCACATTCCCACTGTCAATCACCCGCTCGAGCGACACCAGTGTGGTCGGATGA

>protein

MESFVWFVTLLALASAEKIAFKPCPGDNLGQATSVELSPCPSQPCTFSHGSTVTVVIEFTAVNDSTVLDSRVYGIVETVPVEFPLPNGDGCKDSGIACPLVKGQSYKYTSSFPVLNTYPVISLVVMWKLQAISGNLVCFTFPLSITRSSDTSVVG
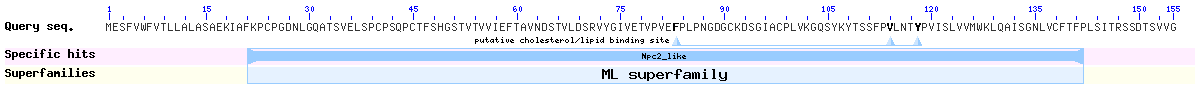


10) Oxysterol-binding protein–related protein 5_NCBI number: PV615410

>mRNA

ATGTCAGGGACCAAGCTATCTAAGAGGGAATCCCTCAAAGAGAAAAAGAAAAGCTACAGAAGCCAAAAGAAAGAAGTGGCCAGAGAGATCATGAACTCAGCCTCCGACCAGTCATTTGTGCTCATGGCCGACTGGTTGAAAGTGAGGGGGACGCTCAAGGGCTGGACCAAACTCTGGTGCGTCTGTAAGCCGGCCATGTTGATCATATACAAGAGTGAGAAGATGAAGACAGGCCACTGGGTGGGGACAATACTGCTCAATACATGCCAACTCCTGCAGAGACCTTCCAAGAAAGAGGGCTTCTGCTTCAAGCTCTTCCACCCGTTGGATCAGTCCATCTGGGCCACTAAAGGACCCAAAGGGGAGACTATTGGGGCCTTTGTCCAACCACTTCCAATGTCCTATGCTATTTTCCGTGCCCCATCAGAGAACCAAGGTAAATGTTGGATGGATGGGCTAGAGTTGGCTTTGCGGTGTACCAGCCTCATCAAGCGAACGACCTCTCGACCCAATGACATTACAAATGACTTATCTTCTGCCTTGACTTCTGACCTCTCACAGACTCTGGTCTCTGACCTGTCCAAGACCCTGGCTCCTCAACACAACACAAGCATGGCTGATGACACCGAGGAGGCTGTGGAGGAGGCAGAGGACCCTCTTGAACATTACAGTGATGATGATGACCACTCCAGTCTAGGTCTAAGTGAATCAGACTTTGAACTGGCAGAGGAGGAGAAAAAACCTCTTGCCATCCCCAAAGTTGAGGAGACCGTTTACACATCTAATGAGGTGGAGGAGCTTGGTCAGCAAGGTGATGCCTGCCAGACAGAGGAGGTGGATGAAGAAAACAAGAGCTTAATTTGGACACTGGTCAAGCAGGTGCGGCCAGGCATGGATCTCTCCAAGGTTGTACTTCCAACTTTTATACTTGAGCCCCGTTCCTTTCTGGACAAACTGACAGACTACTACTACCATGCAGACCTTCTGTCACAGGCAGTCCAACAAGAAACAGCTTTGGAGAGGATGAAGATGGTGGTCAAGTGGTACTTGTCTGGTTTCTACAAGAAACCTAAGGGACTAAAGAAACCCTATAATCCTATCATCGGTGAGACTTTCCGATGTTACTGGGTTCATCCTGAGACCAAAAGTCGGACATTTTATGTAGCAGAACAGATTTCCCATCATCCCCCTATCTCAGCCTTCCATGTCACCAACAGACACGATGGCTTTAACATCAATGGCTGCATTCTGGCCAAGTCCAAGTTTTATGGTAACTCCATCTCAGCTATATTAGAAGGGGTGGCAACCTTGACCTTCTTGGAGAGAGGAGAAGACTACCTTATGACCATGCCATATGCTCACTGCAAAGGAATTCTCCTGGGGACATTAACTATGGAGATGGGTGGGAAAGTGACCATTGACTGTCCTAAGACAGGTTATCATTGTGACCTTGACTTCAAATTGAAGCCCTTTTTTGGCAATGGCGAGGCAGCCAATAGAATCACTGGTCGTATCATCATGGGACAGGAGGTCTTGTGTACCTTTGAAGGCCACTGGGACCAGCAGATCTACATCAAGGAGCTGACCAACAGGGAGAAAGTTCTGTTTTGGGATCCCTCCCCTGAGACCAGGAACAAGCGCTTGGTCAGGTACACAGTGCCGGTAGAGCTGCAGCGTGACAATGAGTCTGAGAGGCTGTGGCGACGTGTCAGTGAAGCCGTGATAGACCAAGACATGCACGCAGCCACAGCTGAGAAACACAAACTTGAAGAGAAGCAGCGCAATGAGGCAAAGGAGCGTCACAAGGTCAAACAAGAGTGGGTGCCCAAATGGTTCTCACTTGACCCCGCCGCCAACAAGTGGATCTACAAACATGTTGATTCCAGACCTTGGGACTCTATGACTGACTGCATACAGTATGAGAAAGATTACATCATAAAGACACAGACAGTACACACAGTTCCACTGGTCTGGAATGCTTCCTCCTGTGTGAACACTGGCCGGATGAAAGCCTTTGGTCCACACTCAACCGTTGTCACAGATGACAGTTACTCATCAGACAACAATGCTGCAGAGTCTGATGATGCTCGCTGTAGAAACACCAACAGGAAATCGAGCCCATCAGAAATTCTCAGTGGTTCTGAAAGTTCAAAGGAGAGAGGCCACCACCATCACCAGGGAGGACCAATTAAAACTGGTCCCAGCAACGAAAACTGGAAGCTGCAGATGCTGCAAGGGGGGGCCCCTGATAGAAAGACAGGGACGCCAGAACATGTTAGACGGCACATGCTATCCACCAACAATCAGAGCAGCAATATAAATGGATTATCTGTTCCTGATAAGGGTGGCCCTTCAAAGGTCAAGGATGACATGAAGTTCACTCAGCATTCACCTGTGAAATGTAACCTCGGGGGTAGCGGGGAGGTCACCCCAATCCCTTCTGACAGCCCCTCCGCCCGACACGCGTCAGCTGGGGTGCACAGCTCATCAAGCAGGACGTCGGTGGGTCAGCACGATGTGCAGATGCTTGTGAAGATCGCCAGGATGCAGCAGGAGATGCAGTCTGTGCTGAACGAGCTCGTCACCAAGGTGGCCAACATCCGGGCCCAGCCTGAGCCCGGTTCACTGGCGCTGTCCCACGGTTATCCCGACAGGGAACAAGCGACCGGGGGCCTGCTGGTGAGAGAATGGATTTTTGTGGCTGTGTTGCTCGTCTTCCAGACTTTGCTGCAGTGGTTCTTCTATAGATCAAGCTGA

>protein

MSGTKLSKRESLKEKKKSYRSQKKEVAREIMNSASDQSFVLMADWLKVRGTLKGWTKLWCVCKPAMLIIYKSEKMKTGHWVGTILLNTCQLLQRPSKKEGFCFKLFHPLDQSIWATKGPKGETIGAFVQPLPMSYAIFRAPSENQGKCWMDGLELALRCTSLIKRTTSRPNDITNDLSSALTSDLSQTLVSDLSKTLAPQHNTSMADDTEEAVEEAEDPLEHYSDDDDHSSLGLSESDFELAEEEKKPLAIPKVEETVYTSNEVEELGQQGDACQTEEVDEENKSLIWTLVKQVRPGMDLSKVVLPTFILEPRSFLDKLTDYYYHADLLSQAVQQETALERMKMVVKWYLSGFYKKPKGLKKPYNPIIGETFRCYWVHPETKSRTFYVAEQISHHPPISAFHVTNRHDGFNINGCILAKSKFYGNSISAILEGVATLTFLERGEDYLMTMPYAHCKGILLGTLTMEMGGKVTIDCPKTGYHCDLDFKLKPFFGNGEAANRITGRIIMGQEVLCTFEGHWDQQIYIKELTNREKVLFWDPSPETRNKRLVRYTVPVELQRDNESERLWRRVSEAVIDQDMHAATAEKHKLEEKQRNEAKERHKVKQEWVPKWFSLDPAANKWIYKHVDSRPWDSMTDCIQYEKDYIIKTQTVHTVPLVWNASSCVNTGRMKAFGPHSTVVTDDSYSSDNNAAESDDARCRNTNRKSSPSEILSGSESSKERGHHHHQGGPIKTGPSNENWKLQMLQGGAPDRKTGTPEHVRRHMLSTNNQSSNINGLSVPDKGGPSKVKDDMKFTQHSPVKCNLGGSGEVTPIPSDSPSARHASAGVHSSSSRTSVGQHDVQMLVKIARMQQEMQSVLNELVTKVANIRAQPEPGSLALSHGYPDREQATGGLLVREWIFVAVLLVFQTLLQWFFYRSS
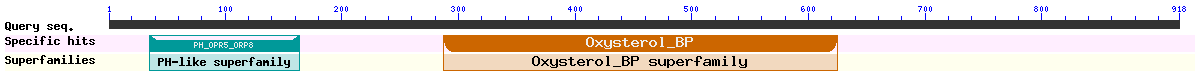


11) Oxysterol-binding protein–related protein 1_NCBI number: PV615411

>mRNA

ATGTCGGAAAATAAATTGGTGGATAATTTGACAACTGTGTGCAGTGATGTTGAGTCGGATGACAGCTCTGAAAGTTTTAACTCTCAAGAGGATGAGCTGTTAAATCTAGCCAGACATGGTAAAGAGGATGCCTTGCTTCAATTCCTACTTACTAATGATGAACGAGACCAGAACCAAAAACTTGACATTAGCTGCAAAGGAAATCAGAAATGTAACAGAGGATGGACAGCACTTCACCTGGCTGCATACTTTGGTCAGACATCTGCTGTGAGATTGTTATTAGAGTTTGGGGCAAGGGTTGATGATTTGAACACAAGCTGTGAAAGCCCTCTGCACCTTGCAGCTTACACTGGTCGTGAGGCAATAGTTGGTTTATTATTACAATATGGGGCTCAGACCAATTTAATCAACAGTCAAGGTCAAACACCTCGTGATGTTACCAAAAATCAACATATTCTTGATATGATAGATGCTGCTAACAGTTCTGAAAGTCTCAGAATCCACTCTGAATTTTTACAGGCTGCAGTTTCAGGTTACACTGAAAAACTGTCTCGGATGTTGAATGATGAAAAAACCAACATTGACGTACAAGACAGCTTTGGCAACACTGCACTCCACATGGCAGCTATGAGGGGTCACGGTGCCGCCTCTGTCATCCTGTTGCAGCATGGAATAGATTCCAGTCTGCGCAACCAGGCTGGGCAAACTGCATTTGATGTAGCAGCCAATCAGAAGATTAAACAAGTTCTAGGGGTTCAATCGATCAGAGCATTACACACTCAGCCAAAGCACTTTCAAGGAATTCTTCTAAAAAAATCTCGATTTGTTGGCTTCAAACCAATGTGGGTTGTCCTAGACAGAGGAGTCCTCTCCTACTTCCTTAACAGGGGAGATGCATCAACCGGCTCCAGAAGAAAAGGAATGAAGTATCTGGACGGAGCTAGAGTGCTGAATTCAAAAGATAAACCAGAAGAAATACGACTAGAATACTCGGACGGCTCAAGACATACACTGCAACTGGCAGAACCAGGCAATAACTCTCCTGTGATATTGCAGAAATGGCTATTTGCCCTTCAAGAGCATATAGCATACAACACCTATTACATTCATCAAGGGGAGGAGACTGACCAGACAGCAAAGGATATGGTCTCCCTTGGCACGATGCAAGAGACACTTCAGAATGCCCAAGCCCACAAGGGGCACCTAGAAAAACAGGTGGAGATCTTACAAGAATCCATTGTTGCCCTCTCCAAACAAATAAGTCAGGACAGCAATGGAGGACCATCAAGGATGGTAACTGCAGCTGACCTGCGGACACTGACCTCACAATCCTCTGATATCCTGAAATCATCTCAGGACATGTTGCAGGCCTTAGGTCACTGTATGGTTCTACTGACCCAGCAAGAAGAGTTGCGTGAGCTCCAGCTGAAAGAAGAACAAGACCGATGCCGTCTGCTTCAAGACTCGCTGCACGCGCTGGCGACGGAGCACCATGAACTAGAGAAGTCGATCACCAGACGTCACTCTTTTCTTTCCATGGACGATGAAGAGTTCTTTGATTGCAACGGTGACAGTATGGAGCACAGAAGTTCACTCGCTATAGGTGATTGTTTAACTGAGAAAAATTCACAAGACTCGTCAAATGATAATGACAAGCAAAATTCAGAGGCCACGCCAAGCTATGGCAGAACCAGGTTACCTGTACCAATGTTTTCTCGATCGGAATTCAGTTTCTGGAGCATTCTGAAACAATGTGTTGGAAAAGAGCTGTCTAAAATTACCATGCCGGTGGTGTTCAATGAACCTCTGAGTTTTATTCAGAGGTTAGCTGAGTACATGGAGTATGCTCCTCTAATTGAGAAAGCTGCCAACTGTGATAACCCAGTGGAACGCATGGAGTTAATCACAGCCTTTGCTATCTCAGCTCTCTCCTCTAATTGGGAAAGAATAGGAAAACCATTCAATCCACTTCTGGGTGAAACTTATGAGTTGGATAGACCAGACCTTGGTTTCCGATTTATGGGAGAGCAGGTCAGCCACCATCCTCCCATCACAGCAATATGTGCAGAAGGCCAGGCCTATGCAATGCATGGCTCTGTGCAGCCAAAGCTCAAGTTCTGGGGCAAAACAGTGGAGATAACCCCTAAAGGACATATATCGTTAGAGCTTAAAAGGTACAATGAGACCTACTCATGGCAAAATGTTAACTGTTGTGTTCACAATGTGATAGTAGGAACTTTATGGGTTGAGCATTATGGCACCATGGAGATTACTTGCAACACCAGTGAAATGAAATCTGTCATCAACTTCAAACAGAGTGGATGGTTTGCTAAAGATCTACATCATGTAGATGGATTTGTTTATAATGGAAAAAAAAAGGAAAGGGCAATATATGGAAGCTGGGTCGCTGGTATTTATTCTTGCAGTCCGAATGAATATGAAAAATTCATGAATGATTCTGAGGCCCAGTCCAAATTTCGGGCCAAGCAAGAGGCTGCCCATGAGAAAAAAGATAAAGATCATCCCAGGTTGTCCATACTGTCTTACAACTATCAACTGGCTGGACAAAAAATTTTGTGGTTGGTCAACCCAAGGTCACCAGAATCTGCTGATTACTACCATTTCTCCATGTTTGCAATGACCCTCAACGAGATGCTCCCAGGAATGCAGCAAGCACTCCCTCCAACAGACTCGAGATTTCGTCCAGATATACGAAACATGGAGGAAGGCAAAATAGATCAGTCAGCTGAGGAAAAAAACAGAGTTGAAGAAAAACAACGTGAAGCGAGGAAAGAAAGGAAAAAGAAAAGCAAAGACTGGCACCCAAGATGGTTCACCTTGAAAAAGATTGGCGGGAGAGAAGACTGGGTGTTCAACCCTGTCTACTGGGATAGAAACTGGAACGAGTGTGCCGACATATTTTGA

>protein

MSENKLVDNLTTVCSDVESDDSSESFNSQEDELLNLARHGKEDALLQFLLTNDERDQNQKLDISCKGNQKCNRGWTALHLAAYFGQTSAVRLLLEFGARVDDLNTSCESPLHLAAYTGREAIVGLLLQYGAQTNLINSQGQTPRDVTKNQHILDMIDAANSSESLRIHSEFLQAAVSGYTEKLSRMLNDEKTNIDVQDSFGNTALHMAAMRGHGAASVILLQHGIDSSLRNQAGQTAFDVAANQKIKQVLGVQSIRALHTQPKHFQGILLKKSRFVGFKPMWVVLDRGVLSYFLNRGDASTGSRRKGMKYLDGARVLNSKDKPEEIRLEYSDGSRHTLQLAEPGNNSPVILQKWLFALQEHIAYNTYYIHQGEETDQTAKDMVSLGTMQETLQNAQAHKGHLEKQVEILQESIVALSKQISQDSNGGPSRMVTAADLRTLTSQSSDILKSSQDMLQALGHCMVLLTQQEELRELQLKEEQDRCRLLQDSLHALATEHHELEKSITRRHSFLSMDDEEFFDCNGDSMEHRSSLAIGDCLTEKNSQDSSNDNDKQNSEATPSYGRTRLPVPMFSRSEFSFWSILKQCVGKELSKITMPVVFNEPLSFIQRLAEYMEYAPLIEKAANCDNPVERMELITAFAISALSSNWERIGKPFNPLLGETYELDRPDLGFRFMGEQVSHHPPITAICAEGQAYAMHGSVQPKLKFWGKTVEITPKGHISLELKRYNETYSWQNVNCCVHNVIVGTLWVEHYGTMEITCNTSEMKSVINFKQSGWFAKDLHHVDGFVYNGKKKERAIYGSWVAGIYSCSPNEYEKFMNDSEAQSKFRAKQEAAHEKKDKDHPRLSILSYNYQLAGQKILWLVNPRSPESADYYHFSMFAMTLNEMLPGMQQALPPTDSRFRPDIRNMEEGKIDQSAEEKNRVEEKQREARKERKKKSKDWHPRWFTLKKIGGREDWVFNPVYWDRNWNECADIF
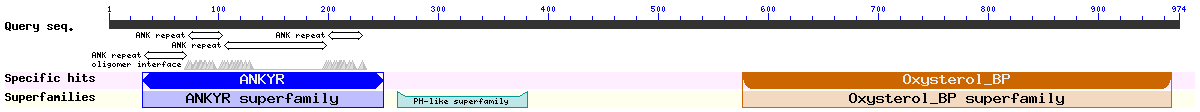


12) VAP protein (both vertebrate VAP-A and VAP-B proteins gave the same hit)_NCBI number: PV615412

>mRNA

ATGGCTAATAAAGAGCAGGCACTTATTTTAGATCCAGCTGGAGAATTACGCTTCAGGGGACCTTTTGTAGATGTTGTAACAGCAAATTTATTATTAACGAACCCTACAGACAAAAGAATATGTTTTAAAGTAAAGACAACGGCACCGAAGCGCTATTGTGTGCGACCTAACAGTGGCATTCTGGATCCCAAAAAGGAAATTGTTGTTGCAGTGATGTTGCAGCCTTTTGACTATGATCCAAATGAAAAAAATAAACACAAGTTCATGGTTCAGTCAATGTTTGCCCCTGATCATATTGGTGATAATCAAGAGATATTGTGGAAAGATGCGCGTCCAGAAAGTCTAATGGATACAAAACTGAAGTGTGTTTTTGAGATGCCGGAGGGGGTCCTTCAACAATCATTGTTATCAGAGACAAGTAAAACTACTGAAGCTATACATTATACATCTGCCATGTCAGATAGCACCTTGGATGACCCAGCAAATGCTAGAAAAGCAGAGTCTCCAAAGAAACCAGCTTCAGCAAGTTCTACAGAGGAAGTAAGGAAACTACAACACGAATTAAAGAAAATACAGCAGGAACTCCAATCACTAAAAAATGCAAATAATAAATTAGAGGAAGAAGGTGTGCGTCTACGCAAGGTAGCAATGTCGGATACCATCATGTCAACTCCTTCACATCCCCCCCCATCATCACCTGTCAGTGTTAGGGCCTTTCCACCTGTGGTATACATTGTTGCTGCGATCCTCCTTGGCCTCATCATCACCTGTCAGTGTTAG

>protein

MANKEQALILDPAGELRFRGPFVDVVTANLLLTNPTDKRICFKVKTTAPKRYCVRPNSGILDPKKEIVVAVMLQPFDYDPNEKNKHKFMVQSMFAPDHIGDNQEILWKDARPESLMDTKLKCVFEMPEGVLQQSLLSETSKTTEAIHYTSAMSDSTLDDPANARKAESPKKPASASSTEEVRKLQHELKKIQQELQSLKNANNKLEEEGVRLRKVAMSDTIMSTPSHPPPSSPVSVRAFPPVVYIVAAILLGLIITCQC
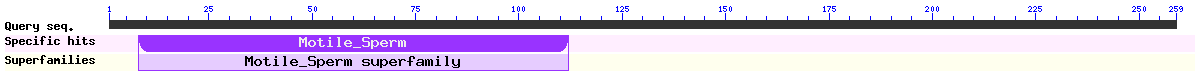


13) Ras-related protein Rab-7a_NCBI number: PV615413

>mRNA

ATGGCATCCAGAAAGAAGGTGCTGTTGAAGGTTATCATATTAGGAGACAGTGGTGTTGGAAAAACTTCTTTGATGAACCAGTATGTCGCCAAGAAATTCAGCAACCAATACAAAGCCACAATAGGAGCAGATTTCCTCACAAAAGAAGTTATGGTGGATGACAGACTAGTCACAATGCAGATCTGGGATACTGCTGGTCAAGAGAGGTTCCAGTCTCTAGGAGTGGCCTTCTATCGTGGCGCTGACGGCTGTGTACTTGTGTTTGATGTGACCATGCCCAACACCTTCCGATCACTGGACAGCTGGAGAGATGAGTTCCTTATTCAGGCATCTCCCAGGGACCCAGAGAACTTTCCCTTTGTTGTTATTGGAAACAAAATTGATTTGGAAAACAGAGCAGTGTCAGCGCGTAGAGCTCAAGGCTGGTGTCACAGTAAAGGAGACATCCCCTACTTTGAAACTTCAGCCAAAGAAGCCATCAACGTGGAGCAGGCCTTCCAGGCAGTGGCCAAGAATGCCATGGCACAAGAGTCTGATGTGGAGCTGTGCAGTGACTTCCCAGACCCCATCAAGCTCAAGGATGATCAAAGCAAGCCAAAGGAAGGGTGTGCATGCTGA

>protein

MASRKKVLLKVIILGDSGVGKTSLMNQYVAKKFSNQYKATIGADFLTKEVMVDDRLVTMQIWDTAGQERFQSLGVAFYRGADGCVLVFDVTMPNTFRSLDSWRDEFLIQASPRDPENFPFVVIGNKIDLENRAVSARRAQGWCHSKGDIPYFETSAKEAINVEQAFQAVAKNAMAQESDVELCSDFPDPIKLKDDQSKPKEGCAC
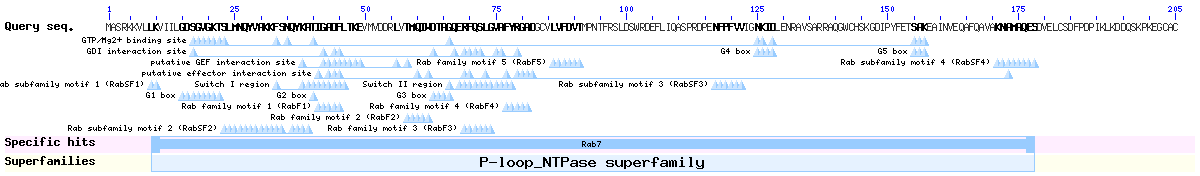


14) Sterol regulatory element-binding protein (both vertebrate SREBPs gave the same hit)_NCBI: PV615414

>mRNA

ATGGCTGACCAACTGAGATGGTCGGTTGGAAATGATAATTTCTCAACATTGGATAATGATTTTCTGTCATCAAATAATTATGAAAATCTTACATTCGACGATTTAGATATAATTCAGTACATTAATGGTGATATCCCAACTGATGGTGACATCTTTAATAACCAAAACTATGACATTGGCATAGAGCAATCACTGGGTTTGGAGACAGATCTGGGCTTGGATCAGCCACTTGGAAATGATCCCAACTTTGGTGATGCAGAATTTAAAGTGGATGAAGTTGGACTTCAACACCAGATATCCTTAGCTGATTCTAAGGTGGAAATTAAACAGGAGCCAAGTTCAATACCATGCCAGTTTGAGCTCCCAGTTACTGGCACTCAAAGTGAACTCACCAGTCTACTTAAACAGGATTTGAAACCCTCTGTACCTCAGGGGTCTAAATCAGTAACAACACATTTGAATATTTTGAATAGTGGACAGCTCTCTCAATCAGCTGCATTGAAAACACTTTTAGAACTACATGATGCTGCTCAAGCAAAACAGATGCAAGAGGCTCAAAAACAACAACAGATCAAACAGCACTTGTCCCAGCAGGCTTCAGCCCAGAGGCTCATCGGTGGATCCATTCAACCAAAGCAGACATCACAGCAACAGCTGAAACTAATTCTACAACAACCTCTCATCAATACCAATGCAAACAACACTAACATTCGTCAGCAGCCCAGCCAGATTCAGACTGTAGCTCCACAAGTCCAAGTCATTCAGCAGCATCCACAGATCAATATTCAGCAGCTTCAGCAGCTGCTGCAGCATGTTGCCAAAGCAAATCAGCCGACTGAACAGCAAAGCAACAGCACCAATGTAATAACCTTGAGTTCAACACCCACATTAAGCACTGTCACCACATCACCATTCCAAACACTTGTTACAGATGGTGGCAATACTATTGTTACTACTAGTATTCCTGTTCAGGTATTTGATGGAGATAAAATGCCAATAAATCGCTTAACAAGTGCACCAAAAATGAAATGTAAAGGAGAGAAGCGTACTGCCCATAATGCCATTGAAAAGCGTTACCGCCTTAGCATCAATGATAAGATTGTTGAGCTTAAAAATCTTGTTGCTGGAGTTGAAGCAAAGTTGAACAAATCTGCTATTCTGCGTAAAGCCATAGACATGATCAGACATCTGCAGAGAGATAATAAGAGATTGAAACAGGAGAATCTCAGTCTTAAAATGATTATCAACAAAACAAATCCACAAGAGCTACTTTTACTAAATGAAAGTGCATCACTTGATGCAGACACCTCAGCCATGACACCACCTTATTCTGACAACTCATCTGGAGCAAACAGCCCCAGTCCTTCTGAAAATGACTGCTCTAATCCCAACAGCCCTGGCAGTGACGACATGATCACTGATGACATTATATATTCTGGGCAATCTGGTATGCTGGATAGATCACGGATCTTTCTGTGCATGTTTATGTTTGGCGTTCTCATTTTCAATCCCTTCAACTATTTCTTCAACTCTAATGCACCATTGGGTGCAGACTCTGGATCTCATCATTCTAGAACTCTCCTTGCAGCTGAATCTGTTTCAAACTGGTATGATAGAATATATCCATCTCTCATACTATGGCTTCTTAATGGCATTTTGGTGAGCTTTGTCCTTGCGAAAGTTCTAATATTTGGAGAACCAGTTACCAAAAAAAATAGTGATGCTTCTGTGGCATTCTGGAGGCACAAAAGGCAAGCAGACCTGGATATGTCAAGAGCAGACTACACATCTGCAAGCAACCAGTTGCGTCAGTGCCTCTTAGCTCTTGGTCGACCATTGCCAACCTCAAAAGTGGATCTACTCTCTGGTGTTGGATGGCAAATCTTTAGGCAGTGCCTTAATCGTCTTTATCTTGGTAGATGGCTGACAAGTCGGGCTGGAAGCCTAACAGGAAATAACAGTGTTGATGTCAAAGAATCTGCAAGAGATGCTGCAAATGTCTTTCATATTTTAAATCAACTACACTTGAGTGGTCACGTGAAAGGCAATCGTCTATGGGGGTTAAACTTAGCTTTGAGTGCTGTTAACATTGGAGAAGCCGCAAAAGATTCTTTGTTGAGATTTGAACTTGCAAGAATATATGCCACTGCTGCGTTACAAATCAAAGCCTCGCTGCCTGAAGGTTTCCAATTTGTTGCCAGATATTTCCTTAGCCGAGCGCGTCACATTTGTAAAAAGAGTGGAGACCAGGTCCCTGCAAATATCCAGTGGCTGTGCCATCCTGAAGGACATCGATTTTTTGTTGACAGTGTTAAGTTTGAGGGTACTGAAGACTCAATTTTTTCATCAAGGGGAACTGAAGTTGATCCTTTGGCTAGAGTGACACAAGCTTTTCGTGAACATTTGCTGGAACAAGCTCTATTTACTCTGGTCTCTCCCAAAGATCCATCTAAAAAAGGAAAACTGGGCGAACCACTTTTATATGCACAACTTCTGTCAGAATGTTCCTGTTTAAGTTCTGGTACTCAAAGCACACATCCAGAAATGGCAGGAATAGCAAAAGTTGGAGAATTAGATGAAGTTGCATTGTGGTGGTCTAACATTGTGTCTGTGGCACATCACTGGCTAACTGGGGATGATGAAAATGCTTCAAGAAACTATTCAGTCTTGGATGTTTTCCCTAAAAAGTTGCATGGAGTTGATGACCCACTTCCAAGAGCAGTGTACTTAGCATACAAAGCCAGGAAAAATGTTTTTGTACAGCCTGATGCAAAGAACTTAAGGGCCTCTATCAGACAGTGTGACAGAGCAGGAAGGCTCTTGAGAGAAAGTCTTAAACTTGCCTACACAGCAGAAAATATCACCATTGTCAAAAGTATTCAACTGCTCCTCTGTGACTGGCTTTTGACAACACGCACTGATATTTGGGAGATTGATAATGAGGAGTGTAATTCAAAATCAGCCTCTCAAACAGAACTTATTGCTTTTCAGCAAGATTTGGGAAGTTTAAGAAAATTATCTCACAGCATGAAAGCAGCATTGCCAAAAGTTTTTCTTCATGAGGCAACCTCAAGAATAATGGCAGGAGCCAGTCCTGGCCGCACACAACAGCTTTTAGATCGAAGTATAAGGCACAGAAATAAACTTTTACAGTCAGAAAAAGATGATTTTGATGTTTTGCTGGATAATGATTCCCAAGAGAGGGAAAGAGCAACAGCTCTGCTTATGGCAGGTCGCCACTTGCCCACCAATATGACAGGGGGTTCCAGTGAAAGAATAAATCTTATTAAAGAAGCTGGTCGCTTGTATGAGGTCCTGGGAGACAAGAAATCTGTTCAGTTGTGTAGGAAAGTCCTCTTGGAAATTGATGAATCAAAGCAAAGCACTGAGGTTCCAGTGGCCGGATGTTAA

>protein

MADQLRWSVGNDNFSTLDNDFLSSNNYENLTFDDLDIIQYINGDIPTDGDIFNNQNYDIGIEQSLGLETDLGLDQPLGNDPNFGDAEFKVDEVGLQHQISLADSKVEIKQEPSSIPCQFELPVTGTQSELTSLLKQDLKPSVPQGSKSVTTHLNILNSGQLSQSAALKTLLELHDAAQAKQMQEAQKQQQIKQHLSQQASAQRLIGGSIQPKQTSQQQLKLILQQPLINTNANNTNIRQQPSQIQTVAPQVQVIQQHPQINIQQLQQLLQHVAKANQPTEQQSNSTNVITLSSTPTLSTVTTSPFQTLVTDGGNTIVTTSIPVQVFDGDKMPINRLTSAPKMKCKGEKRTAHNAIEKRYRLSINDKIVELKNLVAGVEAKLNKSAILRKAIDMIRHLQRDNKRLKQENLSLKMIINKTNPQELLLLNESASLDADTSAMTPPYSDNSSGANSPSPSENDCSNPNSPGSDDMITDDIIYSGQSGMLDRSRIFLCMFMFGVLIFNPFNYFFNSNAPLGADSGSHHSRTLLAAESVSNWYDRIYPSLILWLLNGILVSFVLAKVLIFGEPVTKKNSDASVAFWRHKRQADLDMSRADYTSASNQLRQCLLALGRPLPTSKVDLLSGVGWQIFRQCLNRLYLGRWLTSRAGSLTGNNSVDVKESARDAANVFHILNQLHLSGHVKGNRLWGLNLALSAVNIGEAAKDSLLRFELARIYATAALQIKASLPEGFQFVARYFLSRARHICKKSGDQVPANIQWLCHPEGHRFFVDSVKFEGTEDSIFSSRGTEVDPLARVTQAFREHLLEQALFTLVSPKDPSKKGKLGEPLLYAQLLSECSCLSSGTQSTHPEMAGIAKVGELDEVALWWSNIVSVAHHWLTGDDENASRNYSVLDVFPKKLHGVDDPLPRAVYLAYKARKNVFVQPDAKNLRASIRQCDRAGRLLRESLKLAYTAENITIVKSIQLLLCDWLLTTRTDIWEIDNEECNSKSASQTELIAFQQDLGSLRKLSHSMKAALPKVFLHEATSRIMAGASPGRTQQLLDRSIRHRNKLLQSEKDDFDVLLDNDSQERERATALLMAGRHLPTNMTGGSSERINLIKEAGRLYEVLGDKKSVQLCRKVLLEIDESKQSTEVPVAGC
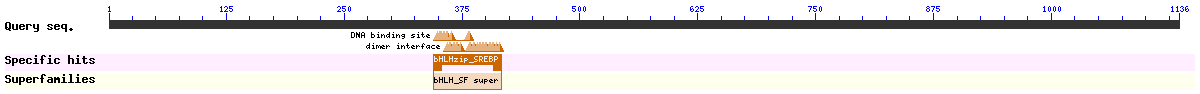


15) Acetyl-CoA acetyltransferase, mitochondrial (acetoacetyl-CoA thiolase)_NCBI number: PV615415

>mRNA

ATGCAGTCGTACCTTAGGATTTCCTCTCAGAGTGTTAAGTCCTTGTTGATGAGTCGGTCCTATGCGTCAGCATCAGGATTGAAAGAGGTAGTCATTGTTAGTGCGGTTCGAACTCCCATAGGATCATTTCTTGGGTCTTTGTCATCTGTCCCTGCCACCAAACTTGGAGCTGCAGCAATCAGTGCAGCAGTTCAAAAAGCAGGAATTGCGCCTGATGTTGTGAATGAGGTGTACATGGGAAATGTTCTTCAGGGAAGACAAGGTCAAGCCCCAAGCCGACAAGCAACTCTTGGTGCTGGTCTTCCCATTACCACCCCAACAACCACAATCAACAAAGTTTGTGCGTCTGGCATGAAAGCCATTATGTTGGCATCACAAAGCCTCATGTGTGGTCACCAGGAAGTTATGGTGGCTGGAGGTATGGAGAGCATGTCTAATGTTCCATTTTATCTCAAGAGAGGAAATCTACCATATGGTGGGATCAACCTAGAGGATGGTATTGTGTTTGATGGACTGACAGATGTATACAACAAATTTCATATGGGAAATTGTGCTGAAAACACAGCAAAGAAATACAAAATCAGCAGAGAAGCACAGGATGAGTTTGCTGTGAGAAGTTACAAACTTAGTCAAAAGTCAGCAGCAGATGGAGTCTTTGCTTCAGAAATCATTTCGGTCGAGGTTCCCCAAAGAAAAGGCGATCCCATTAAAGTGACAGAGGATGAGGAGTATAAGAAAACTGACTTCAGCAAATTTAAGACCCTAAAACCTGCCTTCCAAAAAGAAAATGGCACCGTGACAGCTGCCAATGCCAGCACATTGAATGATGGAGCTGCCGCACTTGTTCTGATGACAGCAGAAGCTGCCAAGAAATATGGGGCCAAACCCTTAGCAAGAGTTGTTGGCTTTGCTGATGCAGCCATTGATCCAATAGATTTCCCTACAGCTCCAGCATTTGCCATTCCAAAGTTATTTGAGAATAGCGGTGTCAAGAAAGAGGATGTTGCCTTGTGGGAAATCAATGAAGCCTTCAGTGTTGTTGTTTTAGCCAATGTTGAAATGCTCAAACTGGACATCAACAAAGTCAATGTTCATGGTGGGGCAGTCAGCATTGGACATCCTATTGGTATGTCTGGTGCCCGCATCACTGGCCACCTGGCACACCATCTGAAATCAGGTGAATATGGTGTAGCAAGTATCTGTAATGGTGGAGGTGGAGCATCTGCCATTTTGATTCAGAAGTTGTAG

>protein

MQSYLRISSQSVKSLLMSRSYASASGLKEVVIVSAVRTPIGSFLGSLSSVPATKLGAAAISAAVQKAGIAPDVVNEVYMGNVLQGRQGQAPSRQATLGAGLPITTPTTTINKVCASGMKAIMLASQSLMCGHQEVMVAGGMESMSNVPFYLKRGNLPYGGINLEDGIVFDGLTDVYNKFHMGNCAENTAKKYKISREAQDEFAVRSYKLSQKSAADGVFASEIISVEVPQRKGDPIKVTEDEEYKKTDFSKFKTLKPAFQKENGTVTAANASTLNDGAAALVLMTAEAAKKYGAKPLARVVGFADAAIDPIDFPTAPAFAIPKLFENSGVKKEDVALWEINEAFSVVVLANVEMLKLDINKVNVHGGAVSIGHPIGMSGARITGHLAHHLKSGEYGVASICNGGGGASAILIQKL


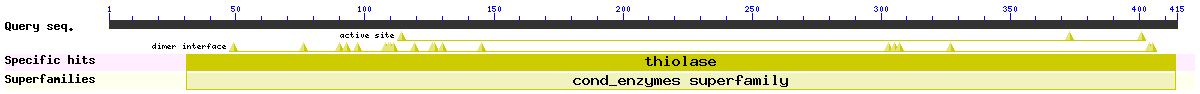


16) Carboxyl Ester Lipase 1_NCBI number: PV615416

>mRNA

ATGATTTTATTTTCAAAATATTTTTTTACAGAAAACATGGCTGTCTTTTACTTCTGCTCACTTTTATTCGCTACGTTCCATTGTTTGTGTCCTGGAGTTTATCCACTCGCCAGCTCCCCAGTGACATTAAACACGAGTCTTGGGCCTGTGACAGGACTTACGAAACGCGTAGCCGGCGTCTACGTTGATATTTTCTACGGCATCCCATTCGCGACTCCTCCTGTTGGAGAGCTCCGCTTCAAACCCCCGGTGCCAGCCCAACCGTGGCGAGACCCACGTGACGTCACACAGAGGCCGAACTCTTGCTGGCAGGTCATTGACACGGCTTTCAATCGCTTCCCTGGGGTTGAGATGTGGAACCCGAACACGCCAATGAGTGAGGACTGCCTGTACCTCAACGTGTGGAGGCCGGCCGGTATCATGTCCAGCGCCAAGCCAATCATGGTGTGGATCTTCGGGGGTAGTTTTCTCACGGGGTCCTCAACACTGGACGTCTACGACCCCTCACAGTTGGCTGCGAGAAATGATGTCATCGTCGTGACCATCGCCTACAGACTCGGAGCTTTGGGATTTCTCTACGCGGGCACTAGTGACGCCCCGGGTAACGCCGGGTTGCTGGATCAGGCGATGGCGCTTAAATGGGTCAAGGACAATGCGGTAACCCTTGGGGGCTCTCCCGATGCCATCACACTGTTTGGCGAGAGTGCCGGGGCCATAAGTATTGGGCTCCACATGGTGTCCCCAGTTTCGAAACACCTTTTCACATACGCCATCATGGAGAGTGGATCACTCTTTCTAACAGGCGTTTTCCGAGATCAGGCTACGGCGAGATCCGGAACGAGGTATCTGGCCAAAACACTCTCTTGTCCTGGAACAGAATTCAGGGACATCGTTGCATGTCTGAGAGTGAAAGACCCGGAAGCGATTGTAAACCAACAGCTTGGCTTCTTTGGTCCTGTCGTGGACGGAACATTTCTTCAAGATGACCCATCCGAGCTGGTCAACAGAGGGGAGATAAAGCCCACCAGCATCTTAACAGGAGTCAACGCCAACGAAGGGAGCTTCTTTGCTGCGTACGAGTTCAAAAATAAGTTCACACTAGACGGAATGGGACATCTGACTGAGAGAGACTATGACGCTTTTCTACGTTCGAGGCATCCCGACGATGCGTCTGCAGTCTCTGAGCTCAATAGAACATATGGGAAAGACCCGACCATTTCGTACGCCGGTCGTGTGGAAGCCATCATTGGGGACGACATGTTTAAATGTCCCGCGGTGAAGTTCGCTCAGCGGTACGCGCCTCTTGGGGAGAATGTCTACCTTTACAGCTTCGAACACAGACTGAGCACCATCCCGTGGCCGCAGTGGATGGGAGTCCCCCACGCATACGAGATTGAGGTCGTCTTTGGCATTCCCTTTATGCCTAACACGAGCTACACGGATGATGAGAGAGAACTGAGTAAGCGAGTGATGACGATGTGGACTAACTTCGCCAAATATGGAAATCCCAATGGTATCGGTAGTGACCAGTGGCCGAGATACACCACCAGAGACCAGAAGTACGTCATCATAGACTCCGGTGAGCTCAGAGTGGCTTCCCATTTGCGACAGCATGAGTGTGGAGCCTTGGCTAGCAGTGGCCAGATGCTTACCTGCCTGTATCTGGTCCTATTGTGGCCTACATACCGGGTCTTTATGGCATTGATGCAAGGGTCAAGTTTACGTTAA

>protein

MILFSKYFFTENMAVFYFCSLLFATFHCLCPGVYPLASSPVTLNTSLGPVTGLTKRVAGVYVDIFYGIPFATPPVGELRFKPPVPAQPWRDPRDVTQRPNSCWQVIDTAFNRFPGVEMWNPNTPMSEDCLYLNVWRPAGIMSSAKPIMVWIFGGSFLTGSSTLDVYDPSQLAARNDVIVVTIAYRLGALGFLYAGTSDAPGNAGLLDQAMALKWVKDNAVTLGGSPDAITLFGESAGAISIGLHMVSPVSKHLFTYAIMESGSLFLTGVFRDQATARSGTRYLAKTLSCPGTEFRDIVACLRVKDPEAIVNQQLGFFGPVVDGTFLQDDPSELVNRGEIKPTSILTGVNANEGSFFAAYEFKNKFTLDGMGHLTERDYDAFLRSRHPDDASAVSELNRTYGKDPTISYAGRVEAIIGDDMFKCPAVKFAQRYAPLGENVYLYSFEHRLSTIPWPQWMGVPHAYEIEVVFGIPFMPNTSYTDDERELSKRVMTMWTNFAKYGNPNGIGSDQWPRYTTRDQKYVIIDSGELRVASHLRQHECGALASSGQMLTCLYLVLLWPTYRVFMALMQGSSLR

**
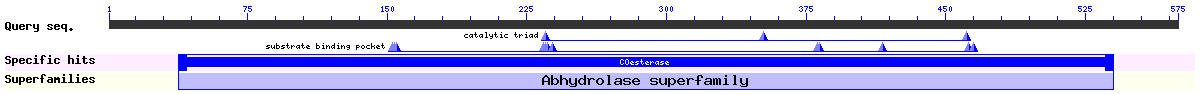
**

**References**

1. Katoh K, Misawa K, Kuma K, Miyata T. MAFFT: a novel method for rapid multiple sequence alignment based on fast Fourier transform. *Nucleic Acid Res* (2002) 30:3059-66. doi: 10.1093/nar/gkf436

2. Capella-Gutierrez S, Silla-Martinez JM, Gabaldon T. trimAl: a tool for automated alignment trimming in large-scale phylogenetic analyses. *Bioinformatics* (2009) 25:1972-3. doi: 10.1093/bioinformatics/btp348

3. Minh BQ, Schmidt HA, Chernomor O, Schrempf D, Woodhams MD, von Haeseler A, et al. IQ-TREE 2: New Models and Efficient Methods for Phylogenetic Inference in the Genomic Era. *Mol Biol Evol* (2020) 37:1530-4. doi: 10.1093/molbev/msaa015
